# TGFB2 mRNA Levels Prognostically Interact with Interferon-Alpha Receptor Activation of IRF9 and IFI27, and an Immune Checkpoint LGALS9 to Impact Overall Survival in Pancreatic Ductal Adenocarcinoma

**DOI:** 10.3390/ijms252011221

**Published:** 2024-10-18

**Authors:** Sanjive Qazi, Vuong Trieu

**Affiliations:** Oncotelic Therapeutics, 29397 Agoura Road, Suite 107, Agoura Hills, CA 91301, USA; vtrieu3@autotelicinc.com

**Keywords:** PDAC, pancreatic cancer, TGF beta, TGFB2, interferon-alpha receptor, RNAseq, mRNA, tumor microenvironment, biomarkers, TAMs, immune checkpoint

## Abstract

The treatment of pancreatic ductal adenocarcinoma (PDAC) is an unmet challenge, with the median overall survival rate remaining less than a year, even with the use of FOLFIRINOX-based therapies. This study analyzed archived macrophage-associated mRNA expression using datasets deposited in the UCSC Xena web platform to compare normal pancreatic tissue and PDAC tumor samples. The *TGFB2* gene exhibited low mRNA expression levels in normal tissue, with less than one TPM. In contrast, in tumor tissue, TGFB2 expression levels exhibited a 7.9-fold increase in mRNA expression relative to normal tissue (*p* < 0.0001). Additionally, components of the type-I interferon signaling pathway exhibited significant upregulation of mRNA levels in tumor tissue, including Interferon alpha/beta receptor 1 (IFNAR1; 3.4-fold increase, *p* < 0.0001), Interferon regulatory factor 9 (IRF9; 4.2-fold increase, *p* < 0.0001), Signal transducer and activator of transcription 1 (STAT1; 7.1-fold increase, *p* < 0.0001), and Interferon Alpha Inducible Protein 27 (IFI27; 66.3-fold increase, *p* < 0.0001). We also utilized TCGA datasets deposited in cBioportal and KMplotter to relate mRNA expression levels to overall survival outcomes. These increased levels of mRNA expression were found to be prognostically significant, whereby patients with high expression levels of either TGFB2, IRF9, or IFI27 showed median OS times ranging from 16 to 20 months (*p* < 0.01 compared to 72 months for patients with low levels of expression for both TGFB2 and either IRF9 or IFI27). Examination of the KMplotter database determined the prognostic impact of TGFB2 mRNA expression levels by comparing patients expressing high versus low levels of TGFB2 (50th percentile cut-off) in low macrophage TME. In TME with low macrophage levels, patients with high levels of TGFB2 mRNA exhibited significantly shorter OS outcomes than patients with low TGFB2 mRNA levels (Median OS of 15.3 versus 72.7 months, *p* < 0.0001). Furthermore, multivariate Cox regression models were applied to control for age at diagnosis. Nine genes exhibited significant increases in hazard ratios for TGFB2 mRNA expression, marker gene mRNA expression, and a significant interaction term between TGFB2 and marker gene expression (mRNA for markers: C1QA, CD74, HLA-DQB1, HLA-DRB1, HLA-F, IFI27, IRF9, LGALS9, MARCO). The results of our study suggest that a combination of pharmacological tools can be used in treating PDAC patients, targeting both TGFB2 and the components of the type-I interferon signaling pathway. The significant statistical interaction between TGFB2 and the nine marker genes suggests that TGFB2 is a negative prognostic indicator at low levels of the IFN-I activated genes and TAM marker expression, including the immune checkpoint LGALS9 (upregulated 16.5-fold in tumor tissue; *p* < 0.0001).

## 1. Introduction

Pancreatic cancer is one of the most devastating types of cancer. It is expected to be the second leading cause of cancer-related mortality by 2030, and its 5-year survival rate is less than 10% [1,2]. More than 50% of pancreatic cancer patients are diagnosed with advanced stages, where the course of the disease is very fast, and the median survival time from the diagnosis is 4–10 months [3]. As for treatment options, surgery is the most common treatment, but unfortunately, only 15–20% of PDAC patients are amenable to resection [4]. Chemotherapy, radiation therapy, immunotherapy, targeted therapy, and palliative care are also used in the treatment of pancreatic cancer. However, the outcomes of combined treatment are still not very promising; aggressive therapy with FOLFIRINOX—a combination of 5FU, Oxaliplatin, Irinotecan, and folic acid—only increases survival to 11 months [3]. Thus, there is an urgent need for novel agents that could be combined with the current standard treatment to improve patients’ overall survival.

**Figure 1:**
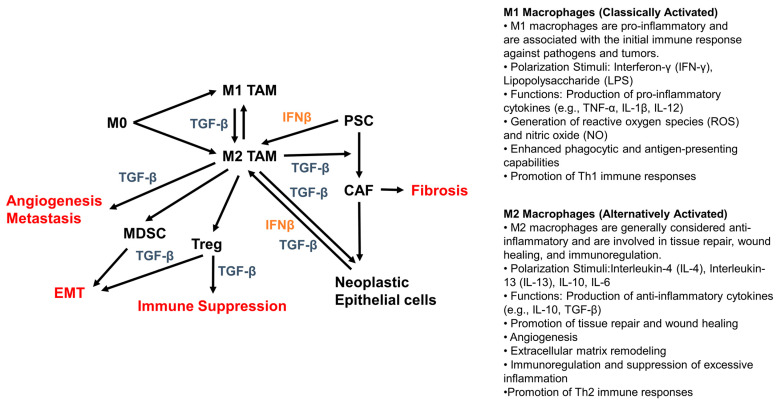
TGF-β and IFN-I pathway impact cellular components of the TME to affect tumorigenesis in PDAC. Once exposed to polarizing stimuli, the undifferentiated or naive state of macrophage (M0) macrophages differentiate into either M1-like or M2-like Tumor-associated macrophages (TAMs). M1 TAMs can undergo polarization via TGF-β to generate M2 TAMs. Subsequently, these M2 TAMs release TGF-β to promote metastasis, angiogenesis, and fibrosis induction by converting PSCs to CAFs. M2 TAMS releases cytokines such as CCL5 and CCL20 to recruit Tregs and releases IL-6, CCL3, CXCL5, and GM-SF to recruit MDSCs into the TME. MDSCs and Tregs promote EMT via TGF-β production [5,6,7,8,9]. IFNβ can be secreted by Neoplastic Epithelial cells and PSCs activate M2 TAMs [8]. Neoplastic Epithelial cells can produce three isoforms of TGF-β and the receptor for these ligands to form a feed-forward activation loop promoting tumorigenic processes [35]. TAM: Tumor-associated macrophage; PSC: Pancreatic Stellate Cells; CAF: Cancer-Associated Fibroblasts; MDSC: Myeloid-Derived Suppressor Cell; Treg: Regulatory T cells; EMT: Epithelial-to-Mesenchymal Transition.

One of the emerging treatment options is targeting Transforming growth factor-β (TGF-β) in the tumor microenvironment (TME). This potent and pleiotropic cytokine plays a complex role in pancreatic cancer. TGF-β orchestrates signaling in a complex ecosystem of cellular components to promote tumor progression (Figure 1). These cellular components include immune cells (Tumor-associated macrophages (TAMs)), regulatory T cells, Cancer-Associated Fibroblasts (CAFs), Myeloid-Derived Suppressor Cells (MDSCs), and Pancreatic Stellate Cells (PSC). Through the action of TGF-β, a diverse set of processes for angiogenesis, metastasis, Epithelial-to-Mesenchymal Transition (EMT), immune suppression, and fibrosis are affected to promote tumor progression [5,6,7,8,9]. TGF-β signaling is one of the 12 core signaling pathways involved in pancreatic cancer [10]. While targeting *TGFβR2*-mutant tumors exposes vulnerabilities to stromal TGF-β blockade in pancreatic cancer, canonical TGF-β signaling suppresses epithelial pancreatic cancer (PDA) cell proliferation, and as a result, inhibiting TGF-β has not been successful [11]. Given the complex role of TGF-β signaling in pancreatic cancer, with both tumor-suppressive and tumor-promoting effects [12], our team has set out to identify the biomarkers associated with this cytokine and the overall survival of patients to establish better combination regimens to target pancreatic cancer cells.

While the full spectrum of key signaling pathways affecting macrophages, ductal cells, and fibroblasts in pancreatic cancer remains elusive, the reports on macrophages show TGF-β signaling contributes to the tumor-enhancing attributes of macrophages in pancreatic ductal adenocarcinoma (PDAC). Tumor-associated macrophages (TAMs) promote PDAC progression by inducing epithelial-to-mesenchymal (EMT) transition [12]. TAMs, myeloid-derived suppressor cells (MDSCs), dendritic cells (DCs), and the TGF-β cytokine are critical in altering the proportion of infiltrating immune cells in PDAC [13]. Macrophages play a key role in PDAC growth and metastasis. In the early stage of cancer, TGF-β reduces the tumorigenic tumor microenvironment by regulating macrophage polarization from M1 to M2 phenotype. However, in the late stage of cancer, TGF-β promotes tumor progression by inducing macrophage polarization from M0/M1 to M2 phenotype (Figure 1) [14]. Vaccination with TGF-β-derived peptides increases the tumoral infiltration of CD8+ T cells. It polarizes TAMs from an M2-like to an M1-like phenotype, which reduces fibrosis and generates a pro-inflammatory TME [15].

Single-cell RNA-seq experiments have further enhanced our understanding of the heterogeneity, functional roles, and metabolic programs of TAMs in pancreatic tumors. These studies have identified novel macrophage subsets, metabolic markers, and signaling pathways contributing to TAM polarization and their interactions within the TME [16,17,18,19,20]. TAMs identified by mRNA molecular markers such as SPP1^+^, C1Q^+^, FCN1^+^, and CCL18^+^ play crucial roles in cancer progression [21]. SPP1⁺ TAMs significantly increase in the tumor microenvironment, are associated with poor prognosis, and promote tumor metastasis and immunosuppression. They secrete cytokines such as VEGFA, PDGF, and angiopoietin, facilitating tumor angiogenesis. Additionally, they interact with endothelial cells to foster angiogenesis and promote EMT, aiding tumor invasion [21]. C1Q⁺ TAMs are involved in immune regulation and immunosuppression, exhibiting high expression of mRNA markers like APOE and TREM2, and are linked with CD8⁺ T cell suppression and dysfunctional immune circuits. These TAMs are found in various cancers and are associated with poor clinical outcomes [21,22]. FCN1⁺ TAMs are inflammatory macrophages associated with tumor-adjacent tissues and angiogenesis. They are derived from monocytes, express markers like FCN1, APOC1, and SPP1, and are precursors of C1Q+ TAMs [20,21,23]. CCL18⁺ TAMs are immunosuppressive and participate in tumor proliferation, angiogenesis, and lymphangiogenesis. They are enriched in M2-like genes and are associated with poor prognosis in neoplastic tumors [24]. CCL18⁺ TAMs function through signaling pathways involving CCL18 mRNA expression, which promotes tumor metastasis by interacting with receptors like PITPNM3 on tumor cells. They also influence EMT and the tumor immune response [21,24,25]. A comprehensive pan-cancer meta-analysis of TAM populations attempted to elucidate molecular signatures for TAM sub-clusters from single-cell RNA-seq studies [26], and based on their signature genes in mouse and human assays, transcription factor profile, and predicated function, the authors classified TAM subsets as interferon-primed TAMs (IFN-TAMs expressing IFN-regulated genes, such as CXCL10, PDL1, and CD86 mRNAs), immune regulatory TAMs (Reg-TAMs expressing ARG1, MRC1, and CX3CR1 mRNAs), inflammatory cytokine-enriched TAMs (Inflam-TAMs expressing IL1B, CXCL1/2//8, CCL3, and CCL3L1 mRNAs), lipid-associated TAMs (LA-TAMs expressing APOC1, APOE, ACP5, and FABP5 mRNAs), pro-angiogenic TAMs (Angio-TAMs expressing VEGFA and SPP1 or other angiogenic factors, such as VCAN, FCN1, and THBS1 mRNAs), RTM-like TAMs (RTM-TAMs expressing LYVE1, HES1, and FOLR2 mRNAs), and proliferating TAMs (Prolif-TAMs expressing MKI67 and CDK1 mRNAs) [26]. Interestingly, examination of TAM sub-clusters from the analysis of treatment-naïve tumors derived from colorectal cancer patients exhibited mRNA upregulation of immune suppressive genes that included TGFB2, CD274, CCL2, and IL10 [27]. In addition, these colorectal TAMs exhibited high expression of MHC and immune co-stimulating genes such as CD80 and CD86, typically markers for M1-like macrophages, suggesting the emergence of TAM with mixed M1/M2 marker expression in TME. Taken together, the answer to the dual action of TGF-β in pancreatic cancer lies within the action of TAMs. It is contingent on the stage of cancer, as a molecularly diverse set of TAMs emerges through the progression of the tumor. Hence, targeting TGF-β and pro-tumor TAMs as a therapeutic strategy for PDAC requires a more complete understanding of their roles in PDAC, especially in the TGF-β interaction with TAM sub-populations in the PDAC tumor.

Another component that is hypothesized to have a crucial role in this crosstalk with TAMs is the function of interferon-alpha (IFN-α), which binds to specific IFN-α receptors (IFNAR) on the surface of macrophages and the formation of Interferon-stimulated gene (ISGF3) complex via IRF9. When activated, IRF9 forms a complex with other transcription factors, such as Signal Transducer and Activator of Transcription 1 (STAT1) and STAT2, to initiate downstream cellular responses. This complex binds to specific DNA sequences and regulates the expression of interferon-stimulated genes (ISGs), which are critical for antiviral defense. This interaction is essential for controlling viral replication and promoting immune defense against viral infections in the normal immune response [28,29,30]. In the context of pancreatic cancer, this pathway mediates radioresistance, inhibits immune responses, and activates survival signaling pathways. Type I Interferon (IFN-I), constitutively produced by cancer cells, sustains prosurvival responses and resistance to DNA-damaging therapies in pancreatic cancer [31]. The IFN-JAK-STAT axis plays a role in bridging radiotherapy to immunotherapy in pancreatic cancer. Conversely, radioresistant pancreatic cancer cells can activate the STAT1-IRF1 axis, which prevents phagocytosis of stressed tumor cells by macrophages [32]. The similarity in the way these components work and the function of TGF-β signaling pathways in cancer, along with established positive crosstalk between IFN-I and TGF-beta signaling, with activation of both pathways observed in preneoplastic rat liver [4], can give us a clue as to how both components can be part of the same core pathway.

A pan-cancer bioinformatic analysis of TGFB2 mRNA showed significant correlations with worse OS and disease-free survival outcomes in PDAC patients analyzed using the TCGA database, and that the TGFB2 mRNA expression levels were also correlated with TAM and cancer-associated fibroblast markers leading to a tumor suppressive and fibrotic TME [33]. Furthermore, as reported in another study, stabilization of TGFB2 mRNA levels by METTL14-mediated m6A modification and then knockdown of TGFB2 mRNA using shRNA found to lower TGFB2 expression decreased gemcitabine resistance in pancreatic cancer cells and increased cell apoptosis. This study found that TGFB2 may contribute to gemcitabine resistance through sterol regulatory element binding factor 1 (SREBF1) and its downstream lipogenic enzymes via PI3K-AKT signaling [34]. Given this prominent role of TGFB2 mRNA expression in PDAC progression and its role in the development of drug resistance, we sought to further clarify the role of TGFB2 mRNA as a prognostic indicator in PDAC patients in the multivariate context with IFN-I activation, as well as gene expression markers for TAMs in the TME. TGFB2 mRNA was a significant negative prognostic indicator in low macrophage environments, suggesting that TGFB2 mRNA levels can impact OS independently of TAMS in the TME. We have hypothesized as such that the function of TGFB2 expression is correlated with Interferon Alpha 1 (IFN-α), possibly through Interferon Regulatory Factor 9 (IRF9) mRNA as a key factor for eliciting the tumorigenic activity of IFN-α, and other members of the STAT family, and targeting these molecules simultaneously can help find more treatment options for pancreatic cancer. The potential exists that different expression levels of IRF family members in different infection and cancer disease settings will determine the level and subtype of IFN-I being produced [28]. Our results suggest that PDAC tumors co-opt this mechanism to impact patient overall survival. Overall, these findings suggest that abrogating IFN-I Interferon (IFN-α: interferon-alpha receptor 1 (IFNAR1), as well as their corresponding downstream signaling molecules, such as STAT1, IRF9, and Interferon Alpha Inducible Protein 27 (IFI27)) could play an important role in successful treatment with TGF-β inhibitors as suggested by the observed impact on overall survival in PDAC patients. Furthermore, TGFB2 mRNA expression levels impact OS independently of 54 out of 81 TAM markers as determined utilizing multivariate Cox proportional hazards models. Nine genes exhibited significant increases in HR for TGFB2 mRNA expression, TAM gene expression, and a significant interaction term between TGFB2 and TAM mRNA expression (C1QA, CD74, HLA-DQB1, HLA-DRB1, HLA-F, IFI27, IRF9, LGALS9, MARCO), suggesting that the impact on OS for TGFB2^high^ group of PDAC patients is modified by the TAM marker gene expression and components of the IFN-I pathway via the expression of IRF9 and IFI27 mRNA, whereby the negative prognostic impact of high TGFB2 mRNA levels becomes more significant at low levels of expression of these nine genes. Notably, LGALS9 mRNA coding for Gal-9 immune checkpoint protein suggests that this protein can be targeted along with TGFB2 mRNA for prognostic and therapeutic purposes. Of these nine genes, the highest levels of expression in tumor tissue were observed for CD74 (Mean ± SEM = 10.94 ± 0.08; 21.8-fold increase relative normal tissue), IFI27 (9.63 ± 0.12; 66.3-fold increase), and HLA-DRA (9.3 ± 0.1; 31-fold increase) for potential use as biomarkers for PDAC prognosis, whereby low levels of expression of these TAM markers would increase the susceptibility of PDAC patients for anti-TGFB2 mRNA directed therapies.

## 2. Results

### 2.1. TGFB2 mRNA Expression and Age Are Significant Negative Prognostic Indicators in PDAC Patients

**Figure 2:**
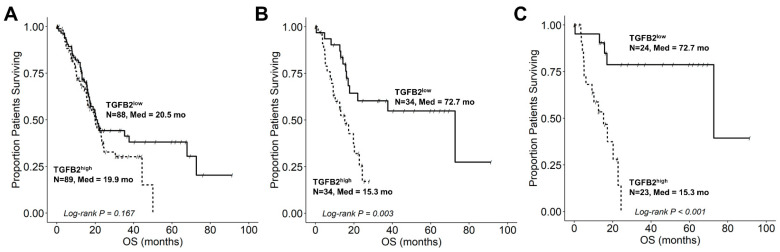
TGFB2 mRNA levels have a significant negative prognostic impact on overall survival (OS) at low macrophage levels in PDAC tumors. OS data correlated with mRNA expression for PDAC patients were depicted as Kaplan–Meier curves to determine the prognostic impact of TGFB2 ligand (TGFB2 median expression cut-off values for high versus low mRNA expression patient sub-groupings) for all PDAC patients ((**A**) N = 177), PDAC patients with low macrophage populations ((**B**) N = 68), and PDAC patients with low macrophage and low neoantigen levels ((**C**) N = 47) (http://kmplot.com/analysis/index.php?p=service&cancer=pancancer_rnaseq, accessed on 23 May 2024). (**A**) The median OS time for 88 patients from the TGFB2^low^ group (20.5 (95% CI: 17.2–NA, Events = 43) months) was not significantly different from the TGFB2^high^ group (N = 89, Median OS = 19.9 (95% CI: 16–24.4, Events = 49) months; Log-rank Chi-Square = 1.91, *p* = 0.167) of PDAC patients. (**B**) PDAC patients with low macrophage burden exhibited a significantly improved OS outcome in the TGFB2^low^ group of patients (N = 34; Median = 72.7 (95% CI: 17.7–NA, Events = 13) months) compared with the TGFB2^high^ group (N = 34; Median = 15.3 (95% CI: 9.3–NA, Events = 21) months; Log-rank Chi-Square = 9.12, *p* = 0.00252). (**C**) In PDAC patients with decreased macrophage and neoantigen levels, the TGFB2^low^ PDAC group (N = 24, Median = 72.7 (95% CI: 72.7–NA, Events = 5) months) experienced significantly longer OS times than the TGFB2^high^ group of patients (Median = 23, Median = 15.3 (95% CI: 9.2–NA, Events = 15) months; Log-rank Chi-Square = 15.66, *p* < 0.001).

We first investigated the potential confounding impact of patient characteristics on the prognostic effect of TGFB2 mRNA levels on OS in PDAC patients. We compared the distribution of patient characteristics: cancer stage, histological grade, sex, and age, partitioned across four sub-groupings based on TGFB2 and IRF9 mRNA expression levels (Figure S1) and TGFB2 and IFI27 mRNA expression levels (Figure S2). A comparison across the four groups suggested a potential confounding impact of histological grade (Gtest of independence, *p* = 0.042) and age at diagnosis (*p* = 0.057) for the four groups of PDAC patients expressing high and low levels of TGFB2 and IRF9 (Figure S1A). However, inclusion of age at diagnosis and histological grade in the multivariate Cox proportional hazards model showed that only age at diagnosis (*p* = 0.031) was a significant independent prognostic indicator for OS (Figure S1B). Similar results were obtained in the patient groupings stratified according to TGFB2 and IFI27 mRNA levels (Figure S2). A comparison across the four groups for TGFB2/IFI27 mRNA expression also suggested a potential confounding impact of histological grade (Gtest of independence, *p* = 0.064) and age at diagnosis (*p* = 0.021) for PDAC patients (Figure 2A). The multivariate Cox proportional hazards model showed age at diagnosis (*p* = 0.049) was a significant prognostic indicator (Figure 2B). Therefore, our investigations only included age at diagnosis in terms of patient characteristics.

We then implemented univariate Cox proportional hazards models to assess the prognostic impact of TGFB ligands (TGFB1/2/3; N = 177), receptors (TGFBR1/2; N = 177), and age (N = 178) on the OS of PDAC patients (Figure S3). There were significant increases in hazard ratio (HR) for the TGFB2^high^ group of patients (HR (95% CI range) = 1.58 (1.03–2.43); *p* = 0.035) and age at diagnosis as a linear co-variate (HR (95% CI range) = 1.0279 (1.007–1.0492); *p* = 0.009) (Figure S3A). Examination of age at diagnosis as a linear covariate with TGFB2^high^ group of patients using the multivariate Cox proportional hazards model showed the HR (95% CI) for TGFB2^high^ group of patients was 1.68 (1.10–2.58; *p* = 0.017). The effect of TGFB2 mRNA was independent of the confounding effect of age at diagnosis for this cohort of PDAC patients (HR (95% CI) was 1.029 (1.009–1.05; *p* = 0.0051)). The Cox proportional hazards model that investigated the interaction of age and TGFB2 mRNA expression was ill-conditioned and over-fitted, with high error bars and low power to detect whether there was a dependency of TGFB2 mRNA levels and age at diagnosis. High levels of TGFB2 expression in PDAC patients exhibited significant increases in HR independent of age, TGFB ligands (Figure S4), and TGFB receptors (Figure S5).

### 2.2. TGFB2 mRNA Levels, but Not TGFB1 and TGFB3 mRNA Levels, Have a Significant Negative Prognostic Impact at Low Macrophage Levels in PDAC Tumors

OS data correlated with mRNA expression for PDAC patients were depicted as Kaplan–Meier curves to determine the prognostic impact of TGFB ligand (TGFB2 median expression cut-off values for high versus low mRNA expression patient sub-groupings) for all PDAC patients, PDAC patients with low macrophage populations, and PDAC patients with low macrophage and low neoantigen levels (Figure 2) The median OS time for 88 patients from the TGFB2^low^ group (20.5 (95% CI: 17.2–NA, Events = 43) months) was not significantly different from the TGFB2^high^ group (N = 89, Median OS = 19.9 (95% CI: 16–24.4, Events = 49) months; *p* = 0.167) of PDAC patients (Figure 2A). PDAC patients with low macrophage burden exhibited a significantly improved OS outcome in the TGFB2^low^ group of patients (N = 34; Median = 72.7 (95% CI: 17.7–NA, Events = 13) months) compared with the TGFB2^high^ group (N = 34; Median = 15.3 (95% CI: 9.3–NA, Events = 21) months; *p* = 0.00252) (Figure 2B). In PDAC patients with decreased macrophage and neoantigen levels, the TGFB2^low^ PDAC group (N = 24, Median = 72.7 (95% CI: 72.7–NA, Events = 5) months) experienced significantly longer OS times than the TGFB2^high^ group of patients (Median = 23, Median = 15.3 (95% CI: 9.2–NA, Events = 15) months; Log-rank Chi-Square = 15.66, *p* < 0.001) (Figure 2C). The negative prognostic impact of TGFB2 mRNA levels observed in this cohort of PDAC patients was not observed for TGFB1 (Figure S6) and TGFB3 (Figure S7). These results suggest that TGFB2 is a specific negative prognostic marker independent of macrophage levels in the TME.

### 2.3. PDAC Tumors Exhibit Augmented mRNA Expression Levels for TGFB Ligands, Interferon-Alpha Receptors, and Downstream Transcriptional Activators to Normal Pancreatic Tissue Samples

Since TGFB ligands and receptors and interferon-alpha pathways can both activate macrophages in the TME, we utilized RNA sequencing (RNAseq) data files that summarize log_2_ transformed transcripts per million values (TPM) to perform a comparative analysis of gene expression levels in 178 PDAC patients and normal pancreatic tissue samples (Table S1). TGFB2 exhibited very low expression levels in normal tissue (<0 log2 TPM equivalent to TPM value of 1) but experienced a significant increase in expression levels in tumor tissue: mean ± SEM log2 transformed TPM values for TGFB2 mRNA expression was found to be significantly higher in PDAC patients (2.76 ± 0.11) compared to normal tissue (−0.22 ± 0.12). The median and range of TGFB2 mRNA expression was −0.09 (−9.97–3.21) for normal tissue and 2.82 (−3.05–6.54) for PDAC patients, representing a 7.9-fold increase in mRNA expression relative to normal tissue (*p* < 0.0001). Through gene expression level analysis, it was discovered that IFI27, the downstream product of IRF9/STAT1 transcriptional activation by IFNAR1, exhibited a significant increase in expression in tumor tissue compared to normal tissue, with a 66.3-fold increase in mRNA expression relative to normal tissue (*p* < 0.0001). IFI27 displayed the highest-fold change in all the genes examined relative to normal tissue and one of the most abundant genes in tumor tissue. In normal tissue, the average expression level of IFI27 was 3.58 ± 0.13, while in patients with PDAC, it was 9.63 ± 0.12. The median expression level and range were 3.66 (−9.97–8.36) for normal tissue and 9.78 (4.53–12.46) for PDAC patients. Components of the type-I interferon signaling pathway exhibited significant upregulation of mRNA levels in tumor tissue: Interferon alpha/beta receptor 1 (IFNAR1; 3.4-fold increase, *p* < 0.0001), Interferon regulatory factor 9 (IRF9; 4.2-fold increase, *p* < 0.0001), and Signal transducer and activator of transcription 1 (STAT1; 7.1-fold increase, *p* < 0.0001) (Table S1).

### 2.4. Patients with Low Levels of TGFB2 and the Transcription Factor, Interferon Regulatory Factor 9 (IRF9), or the Transcriptional Product, Interferon Alpha Inducible Protein 27 (IFI27) mRNA Expression Had Improved OS Times Compared to Those with High Levels of Either TGFB2, IRF9, or IFI27

**Figure 3:**
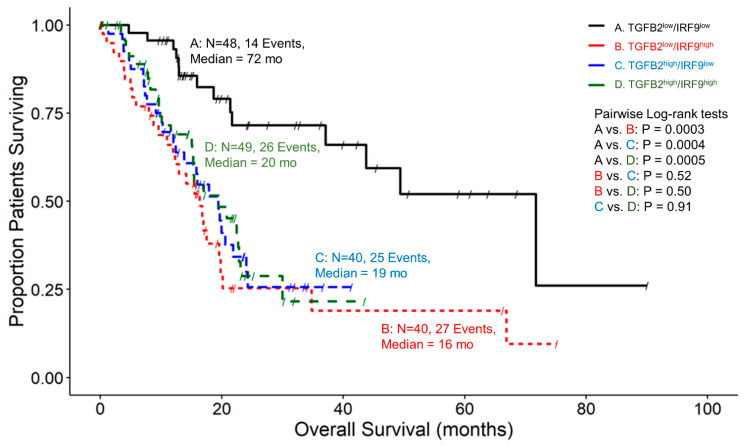
PDAC patients with low levels of TGFB2 and low levels of IRF9 mRNA expression exhibited improved OS times than patients with high levels of TGFB2 or IRF9 mRNA expression. The median survival times for four different groups of patients are as follows: for 48 patients in group TGFB2^low^/IRF9^low^, the median survival time was 72 months (95% CI: 43.8–NA, Events = 14); for 40 patients in group TGFB2^low^/IRF9^high^, the median survival time was 16 months (95% CI: 12–20.2, Events = 27); for 40 patients in group TGFB2^high^/IRF9^low^, the median survival time was 19 months (95% CI: 13.8–24.3, Events = 25); and, for 49 patients in group TGFB2^high^/IRF9^high^, the median survival time was 20 months (95% CI: 15.1–30, Events = 26). TGFB2^low^/IRF9^low^ group of patients exhibited significantly longer survival times compared to all combinations of TGFB2 and IRF9 expression levels (*p*-value = 0.0003, 0.0004, and 0.0005 for TGFB2^low^/IRF9^high^, TGFB2^high^/IRF9^low^ and TGFB2^high^/IRF9^high^ respectively).

**Figure 4:**
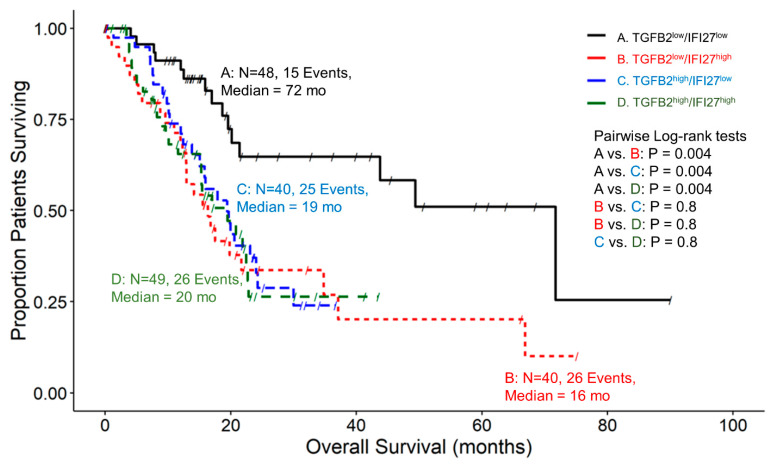
PDAC patients with low levels of TGFB2 and low levels of IFI27 mRNA expression exhibited improved OS times than patients with high levels of TGFB2 or IFI27 mRNA expression. Results of the OS examination for each group are as follows: 48 patients from TGFB2^low^/IFI27^low^ had a median survival time of 72 months (95% CI: 21.4–NA, Events = 15). 40 patients from TGFB2^low^/IFI27^high^ had a median survival time of 16 months (95% CI: 12.9–37.1, Events = 26). 40 patients from TGFB2^high^/IFI27^low^ had a median survival time of 19 months (95% CI: 15.1–30, Events = 25). Finally, 49 patients from TGFB2^high^/IFI27^high^ had a median survival time of 20 months (95% CI: 15.1–22.8, Events = 26). TGFB2^low^/IFI27^low^ group of patients exhibited significantly longer survival times compared to all combinations of TGFB2 and IFI27 expression levels (*p*-value = 0.004 for all comparisons).

TGFB2^low^/IRF9^low^ group of patients exhibited significantly longer survival times (Median = 72 months) compared to all combinations of TGFB2 and IRF9 expression levels (Median = 16, 19, 20 months, corresponding to *p*-values of 0.0003, 0.0004, and 0.0005 for TGFB2^low^/IRF9^high^, TGFB2^high^/IRF9^low^, and TGFB2^high^/IRF9^high^, respectively) (Figure 3). Similar results were obtained examining TGFB2 and the IRF9 transcriptionally activated product, IFI27, in PDAC patients (Figure 4). The patients with low levels of both genes had significantly longer survival times compared to all combinations of TGFB2 and IFI27 expression levels (*p*-value = 0.004 for all comparisons) (Figure 4).

### 2.5. TGFB2 and IFN-I Activated Pathway Gene Expression Exhibited Independent Increases in Hazard Ratios Using Multivariate Cox Proportional Hazards Models

**Figure 5:**
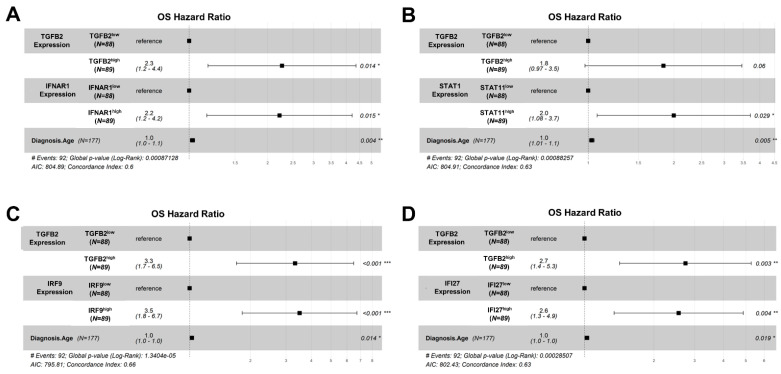
PDAC patients with high levels of IFNAR1/STAT1/IRF9/IFI27 mRNA expression exhibited significantly increased hazard ratios in the multivariate Cox proportional hazards model considering age and TGFB2 levels. Multivariate analyses of the potential prognostic impact of TGFB2 mRNA expression levels paired with IFNAR1 (**A**), STAT1 (**B**), IRF9 (**C**), and IFI27 (**D**) mRNA expression levels on OS were determined using the multivariate Cox proportional hazards model to adjust for age, and TGFB2 statistical interaction with IFNAR1/STAT1/IRF9/IFI27. All four models exhibited significant concordant values (0.6–0.66, *p* < 0.001 for all models), suggesting a good fit of the models to the OS data. High levels of TGFB2 exhibited significant increases in HR for models with IFNAR1 (HR (95% CI range) = 2.27 (1.18–4.36); *p* = 0.014), IRF9 (HR (95% CI range) = 3.32 (1.71–6.47); *p* < 0.001) and IFI27 (HR (95% CI range) = 2.74 (1.42–5.28) and *p* = 0.003). IFNAR1 (HR (95% CI range) = 2.22 (1.17–4.22); *p* = 0.015), STAT1 (HR (95% CI range) = 2 (1.08–3.71); *p* = 0.029), IRF9 (HR (95% CI range) = 3.5 (1.82–6.72); *p* < 0.001) and IFI27 (HR (95% CI range) = 2.56 (1.34–4.87) and *p* = 0.004) showed significant increases in models that included TGFB2 expression controlling for age at diagnosis and interaction terms. *** indicates *p* < 0.01, ** indicates *p* < 0.01, and * indicates *p* < 0.05. # refers to the number of patients experiencing death events.

Next, we tested whether the impact of TGFB2 mRNA expression was independent of the interferon-alpha pathway. Multivariate analyses of the potential prognostic impact of IFNAR1/STAT1/IRF9/IFI27 mRNA expression levels on OS were determined using the multivariate Cox proportional hazards model adjusted for age and TGFB2 statistical interaction with IFNAR1/STAT1/IRF9/IFI27 (Figure 5, Table S2). The Cox multivariate proportional hazards model examining the effects of TGFB2 and IFNAR1 mRNA expression levels showed that there was a significant increase in HR for the TGFB2^high^ group of patients (HR (95% CI range) = 2.27 (1.18–4.36); *p* = 0.014). In addition, there was a significant increase in HR for the IFNAR1^high^ group of PDAC patients (HR (95% CI range) = 2.22 (1.17–4.22); *p* = 0.015), accounting for the significant effect of age at diagnosis in this model (HR (95% CI range) = 1.03 (1.01–1.05); *p* = 0.004). There was no significance of the interaction term in the model (HR (95% CI range) = 0.51 (0.22–1.2); *p* = 0.122) (Figure 5A). Examination of the model investigating the statistical interaction between STAT1 and TGFB2 showed that there was a borderline significant increase in HR for the TGFB2^high^ group of patients (HR (95% CI range) = 1.84 (0.97–3.46); *p* = 0.06) There was a significant increase in HR for the STAT1^high^ group of patients (HR (95% CI range) = 2 (1.08–3.71); *p* = 0.029), accounting for significant impact of age at diagnosis (HR (95% CI range) = 1.03 (1.01–1.05); *p* = 0.005). The interaction term in the model was not found to be significant (HR (95% CI range) = 0.7 (0.3–1.63); *p* = 0.413) (Figure 5B). This model was also used to examine the statistical interaction between IRF9 and TGFB2 and showed a significant increase in the hazard ratio for patients in the TGFB2^high^ group of patients. The group of patients with high TGFB2 expression showed a significant increase in HR (HR (95% CI range) = 3.32 (1.71–6.47); *p* < 0.001). Similar results were found for the IRF9^high^ group of patients (HR (95% CI range) = 3.5 (1.82–6.72); *p* < 0.001), controlling for the significant effect of age (*p* = 0.014) and the interaction term (*p* = 0.002) (Figure 5C). The results showed that patients in the TGFB2^high^ group had a significant increase in HR (with HR (95% CI range) = 2.74 (1.42–5.28) and *p* = 0.003) when analyzed paired with IFI27 mRNA expression. Similarly, patients in the IFI27^high^ group also had a significant increase in HR (with HR (95% CI range) = 2.56 (1.34–4.87) and *p* = 0.004), controlling for significant effects of age and the interaction term (*p* = 0.019 and *p* = 0.032 respectively) (Figure 5D, Table S1).

### 2.6. TGFB2 and Macrophage Marker Gene Expression Exhibited Independent Increases in Hazard Ratios Using Multivariate Cox Proportional Hazards Models

**Figure 6:**
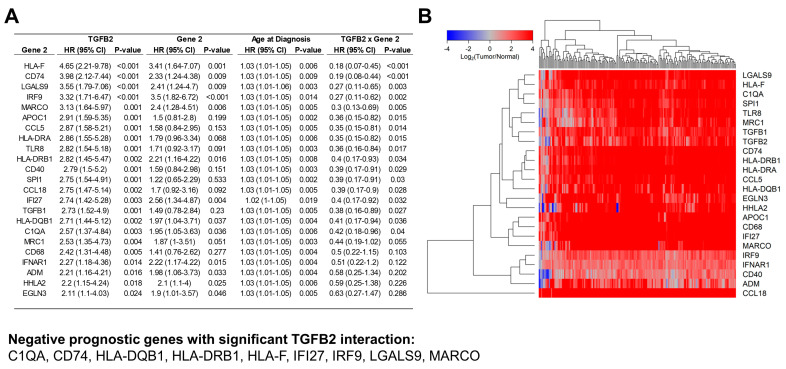
Comparison of normal versus tumor tissue samples for genes exhibiting significant prognostic impacts on OS from the multivariate Cox regression models. Multivariate analyses of the potential effect of TGFB2 in combination with macrophage markers (CD68 (M1), MRC1/CD206 (M2), and 17 identified from single-cell RNA seq experiments), TGFB1, IFNAR1, IFI27, and IRF9 mRNA levels on OS were determined using the multivariate Cox proportional hazards model adjusted for age. (**A**) This Table shows the calculated HRs for (i) The mRNA expression level for TGFB2 as a categorical variable comparing high (N = 89) versus low (N = 88) TGFB2 mRNA expression levels (50% cut-off for the range of TPM values), (ii) The mRNA expression level for the companion gene, Gene 2, as a categorical variable comparing high (N = 89) versus low (N = 88) Gene 2 mRNA expression levels (50% cut-off for the range of TPM values), (iii) age at diagnosis as a linear covariate and an interaction term (TGFB2 x Gene2) included in the model. This Table is ordered according to the descending order of TGFB2 expression impact on OS. Gene 2 refers to identifying the paired gene mRNA expression with TGFB2 mRNA levels in the leftmost column. The impact on OS hazard ratio (HR) for Gene 2 is indicated in the 3rd column, and the interaction is reported in the 5th column. Nine genes exhibited significant increases in HR for TGFB2 expression, Gene 2 expression, and a significant interaction term (C1QA, CD74, HLA-DQB1, HLA-DRB1, HLA-F, IFI27, IRF9, LGALS9, and MARCO mRNAs). (**B**) We compared gene expression levels reported for 178 PDAC patients versus 167 pancreatic tissue samples. This resource reports results from the UCSC Toil RNAseq recompute compendium, which is a standardized realigned and recalculated gene and transcript expression dataset for all TCGA and GTEx samples that enables users to contrast gene and transcript expression between TCGA “tumor” samples and corresponding GTEx “normal” samples. Depicted is a cluster figure of the mRNA expression levels for Macrophage markers, TGFB1/2, IFNAR1 activated receptors and the corresponding downstream signaling molecules (IRF9), and the transcriptional product, Interferon Alpha Inducible Protein 27 (IFI27) mean centered to the corresponding mRNA expression levels in normal tissue. The cluster figure shows the log2-transformed fold-change values (blue represents underexpression, and the red color represents overexpression in samples from PDAC patients). Examination of the dendrogram showed that the expression of CCL8 mRNA was distinct from the mRNA expression of other genes and IFNAR1 was co-regulated with ADM, CD40, and IRF9. IFI27 mRNA was coregulated with MARCO, CD68, and APOC1. TGFB2 was co-regulated with TGFB1, MRC1/CD206, TLR8, SPI1, C1QA, HLA-F, and LGALS9 mRNA. LGALS9 presents itself as a potential target to develop immune checkpoint inhibitors against the production of Gal-9 protein in PDAC TME as it exhibits a 16.5-fold increase in tumor tissues (*p* < 0.0001).

We examined TGFB2-macrophage marker pairs that exhibited either significant increases in both TGFB2 and macrophage marker OS HRs or an increase in TGFB2 hazard ratio and a significant (*p* < 0.05) interaction effect, revealing 21 gene markers that impact OS in combination with TGFB2 levels (17 markers from single-cell RNA seq experiments, TGFB1, IFI27, IFNAR1, and IRF9) (Figure 6). The M1 marker, CD68, only exhibited a significant independent effect from TGFB2 (HR (95% CI) = 2.42 (1.31–4.48), *p* = 0.005), and no effect of CD68 (HR (95% CI) = 1.41 (0.76–2.62), *p* = 0.277). The M2 marker, MRC1/CD206, showed a highly significant impact for TGFB2 in this multivariate context (HR (95% CI) = 2.53 (1.35–4.73), *p* = 0.004) and a borderline significant impact for MRC1/CD206 (HR (95% CI) = 1.87 (1–3.51), *p* = 0.051). TGFB2 exhibited hazard ratios of greater than 3 analyzed in the multivariate model with the following genes: CD74 (HR (95%CI) = 3.98 (2.12–7.44)); HLA-F (HR (95%CI) = 4.65 (2.21–9.78)); IRF9 (HR (95%CI) = 3.32 (1.71–6.47)); LGALS9 (HR (95%CI) = 3.55 (1.79–7.06)); and MARCO (HR (95%CI) = 3.13 (1.64–5.97)). One of the macrophage markers also showed HR greater than 3: HLA-F (HR (95%CI) = 3.41 (1.64–7.07)). IRF9 exhibited an independent increase in HR (HR (95%CI) = 3.5 (1.82–6.72)). Nine genes exhibited significant OS HR impacts with significant effects on TGFB2, Gene 2 with significant interaction effects suggesting co-dependency of Gene 2 on TGFB2 levels (C1QA, CD74, HLA-DQB1, HLA-DRB1, HLA-F, IFI27, IRF9, LGALS9, MARCO) (Figure 6A). We depicted gene expression levels for 178 PDAC patients versus 167 pancreatic tissue samples using a cluster figure of the mRNA expression levels for macrophage markers, TGFB1/2, interferon-alpha receptor 1 (IFNAR1) activated receptors, and the corresponding downstream signaling molecules (IRF9) and the transcriptional product, Interferon Alpha Inducible Protein 27 (IFI27) mean centered to the corresponding mRNA expression levels in normal tissue. All 24 genes exhibited fold changes greater than 2 (*p* < 0.0001 for all comparisons) (Table S3). Examination of the dendrogram showed that the expression of CCL8 was distinct from the other genes, IFNAR1 was co-regulated with ADM, CD40, and IRF9. IFI27 was coregulated with MARCO, CD68, and APOC1. TGFB2 was co-regulated with TGFB1, MRC1/CD206, TLR8, SPI1, C1QA, HLA-F, and LGALS9 (Figure 6B). Sixteen of the macrophage markers were upregulated greater than 10-fold in the tumor compared to normal tissues: CCL18, MARCO, APOC1, CD68, HLA-DRA, HLA-DQB1, CD74, CCL5, HLA-DRB1, EGLN3, C1QA, LGALS9, HHLA2, HLA-F, TLR8, and SPI1 mRNA. Of these 16 genes, TLR8 mRNA was expressed at very low levels in tumor tissue (<1 TPM). TGFB2, MRC1, MARCO, CCL8, CCL5, and EGLN3 mRNAs were expressed at very low levels in normal tissues. The highest levels of expression in tumor tissue were observed for CD74 (Mean ± SEM = 10.94 ± 0.08), IFI27 (Mean ± SEM = 9.63 ± 0.12), and HLA-DRA (9.3 ± 0.1) (Table S3, Figure S8).

**Figure 7:**
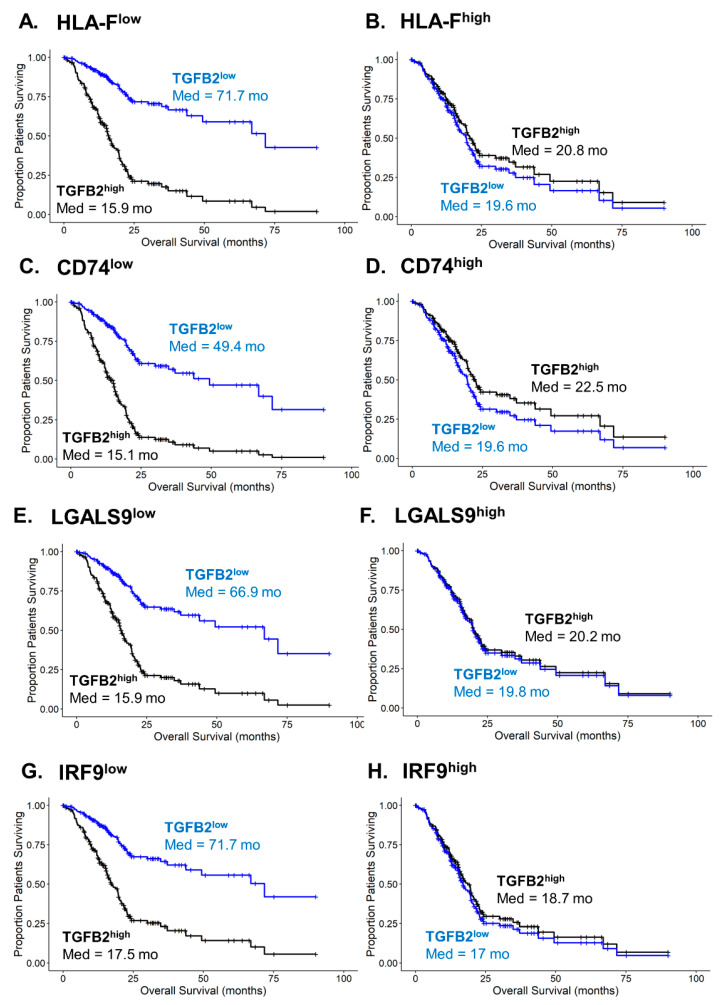
Simulation of OS curves calculated from the multivariate Cox proportional hazards models demonstrating the prognostic impact of TGFB2 mRNA levels at low levels of mRNA, TAM markers, and IRF9. The predicted survival proportion was calculated from the Cox proportional hazards regression model parameters that included the statistical interaction term for combinations of TGFB2^high^ (black line) versus TGFB2^low^ (blue line) groups of PDAC patients in the context of the macrophage marker groups’ high and low mRNA expression (mean diagnosis age for this cohort of patients was set at 64.5 years to run the models for N = 177 and 92 death events for each model (**A**–**H**)). The four most significantly impacted increases in HR for the effect of high levels of TGFB2 mRNA are presented for the paired analysis (see Figure 5) with HLA-F (**A**,**B**), CD74 (**C**,**D**), LGALS9 (**E**,**F**), and IRF9 (**G**,**H**). These four macrophage markers exhibited significant interaction effects in the multivariate model in which the negative prognostic effect of high TGFB2 levels occurred at low levels of the macrophage marker. The impact of the significant interaction term in the models is presented as improvements in OS at low levels of TGFB2 mRNA when there are low levels of the paired marker genes (**A**,**C**,**E**,**G**). At high TAM markers and IRF9 levels, the TGFB2^low^ group of PDAC patients did not exhibit improvements in median OS (**B**,**D**,**F**,**H**).

Calculation of predicted survival curves used the Cox proportional hazards regression model parameters that included the interaction term for combinations of TGFB2^high^ versus TGFB2^low^ groups of PDAC patients in the context of the macrophage marker groups’ high and low mRNA expression (Figure 7). The four most significantly impacted increases in HR for the effect of high levels of TGFB2 mRNA included HLA-F, CD74, LGALS9, and IRF9. The significant interaction term in the models shows improvements in overall survival (OS) at low levels of TGFB2 mRNA when paired with low levels of Gene2 (Figure 7A,C,E,G; predicted median OS times ranged from 49.4 to 72.7 months for TGFB2^low^/Gene2^low^ group of PDAC patients compared to median OS times ranging from 15.1 to 17.5 months for the TGFB2^high^/Gene2^low^ group of patients). Conversely, at high levels of marker gene Gene2 mRNA levels, all combinations of TGFB2 and marker gene subgroups exhibited short OS times whereby the TGFB2^low^ group of PDAC patients did not exhibit improvements in median OS compared to the TGFB2^high^ group of patients (Figure 7B,D,F,H; predicted OS times ranged from 17 to 22.5 months).

## 3. Discussion

### 3.1. A High Level of TGFB2 mRNA, Not TGFB1 or TGFB3 mRNA, Is a Negative Prognostic Marker for OS in PDAC Patients

Our studies directly examined the isoform-specific prognostic impact of TGFB1/2/3 on the OS of PDAC patients. These analyses examining mRNA levels in PDAC tumors indicate that the Transforming Growth Factor Beta 2 (TGFB2) gene exhibited low expression levels in normal tissue, with less than 0 log2 TPM (equivalent to a TPM value of 1). In contrast, in tumor tissue, TGFB2 mRNA expression levels are significantly higher, representing a 7.9-fold increase in mRNA expression relative to normal tissue (*p* < 0.0001) (Table S1). An increase in expression was also observed for TGFB1 (8.3-fold increase; *p* < 0.0001) and TGFB3 (2-fold increase; *p* < 0.0001) mRNA expression levels (Table S1). Examination of the impact of TGFB1/2/3 mRNA levels on OS using univariate Cox proportional hazards models showed that only TGFB2 mRNA exhibited a significant increase in hazard ratio (HR) (Figure S3A). Age at diagnosis was a potentially confounding factor in the TGFB2 impact on OS, as the univariate Cox proportional hazards model showed a significant increase in HR (*p* = 0.009). A multivariate model that controlled for age at diagnosis maintained a significant effect on hazard ratio (HR) for high levels of TGFB2 in PDAC patients (HR = 1.68 (1.10–2.58; *p* = 0.017)). The impact of TGFB2 was also independent of TGFB1 or TGFB3 levels in the multivariate models that controlled for age (Figure S4).

As cancer progresses, TGFB pathways facilitate epithelial-to-mesenchymal transition (EMT), activating cancer cells to acquire mesenchymal and stem cell properties. This allows them to dissociate from the primary tumor mass, invade surrounding tissue, and intravasate into blood vessels [12]. In mouse genetic models, the expression of oncogenic *KRAS* in pancreatic or acinar cells is closely associated with the development of acinar-to-ductal metaplasia (ADM) and low-grade pancreatic intraepithelial neoplasia due to interactions with various macrophage populations and TGFB ligands [5]. M1-like macrophages contribute to ADM by secreting cytokines/chemokines such as TNF, CCL5, IL-6, and IL-1α, or through Matrix metalloproteinases (MMPs), which is followed by the release of IL-13 by DCLK1+ cells and duct-like cells that lead to the polarization of M1-like to M2-like macrophages. Additionally, macrophage release of TGFB ligands induces fibrosis by activating pancreatic stellate cells [5,6,36,37,38].

Cross-talk of TGFB pathway and EMT-associated pathways related to stem cell properties have been characterized by utilizing single-cell RNA-seq data showing upregulation of mRNAs encoding factors involved in BMP signaling, PI3K-AKT signaling, RAS signaling, KIT signaling, ERKs signaling, YAP/TAZ, HIPPO, NOTCH, and Wnt pathways [39], suggesting a widespread impact of TGFB pathways in the TME. Preclinical mouse studies have demonstrated TGF-β2’s specific impact on PDAC growth and development: knockdown of TGF-β2-neutralizing antibodies reduced PDAC invasion of BxPC3 or SW1990 cells measured using the Transwell invasion assay [40]; TGF-β2 mRNA was found to be a target for the micro RNA, miR-141-3p, negatively regulating TGF-β2 expression in PNCA-1 cells; XIST attenuated the inhibition of TGF-β2 expression by miR-141-3p, resulting in proliferation, migration, and invasion of pancreatic cell lines [41]; miR-132 also inhibits TGFB2 mRNA expression by binding to the 3′UTR region to inhibit tumor growth, and this effect is reversed by Dexamathosone (DEX) promoter hypermethylation of the micro RNA gene resulting in DEX-induced clonogenicity, migration and proliferation of AsPC-1, PANC-1 and ASAN-PaCa cells, suggesting a mechanism for the induction therapy resistance via TGFB2 mRNA expression [42]; a study examining the role of Snail (Snai1) in pancreatic cancer transgenic mouse model expressing Snail and Kras^G12D^ exhibited TGF-β2-specific increases in collagen production by pancreatic stellate cells [43]; TGF-β2 was demonstrated to promote endothelial to mesenchymal transition in murine pancreatic microvascular endothelial cells, and reduction in Snail using CRISPR)/CRISPR-associated protein 9 (Cas9)-mediated gene editing counters this phenomenon [44]; in a humanized mouse model, it was demonstrated that inhibiting TGF-β2 production by TGF-β2 antisense oligonucleotide combined with IL-2 augmented T-cell mediated anti-tumor immunity against *SMAD4*-mutated PDAC via tumor-associated fibrosis [45]; using a TGF-β2-blocking antibody, it was shown that TGF-β2 contributed to the HOXA10-promoted invasion and migration of pancreatic cancer cells [46].

These results suggest that the specific targeting of TGFB2 mRNA, not TGFB1 or TGFB3, will derive the most therapeutic benefit in PDAC patients. The prognostic impact of TGFB2 mRNA levels on OS was enhanced in patients with low macrophage abundance (Figure 2), suggesting a significant clinical outcome from statistical interactions between TGFB pathways and TAMs in the TME. The OS outcomes for PDAC patients appear to have both TGFB2-macrophage-dependent and independent mechanisms in the TME.

### 3.2. IFN-I Response Is a Negative Prognostic Indicator in PDAC Patients and Impacts the Effect of TGFB2 mRNA Levels on OS Outcome

**Figure 8:**
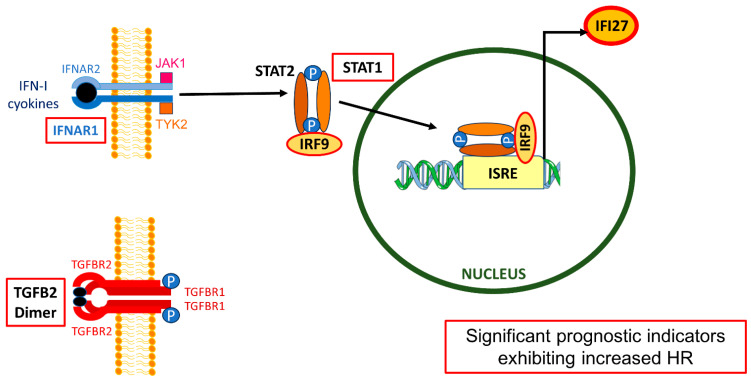
TGFB2 and components of the Interferon type I response mRNA levels prognostically impact OS outcomes in PDAC patients. This Figure illustrates a simplified model for the Interferon type-I (IFN-I) stimulated gene (ISG) transcription mediated by STAT1/STAT2/IRF9 complexes. IFN-I is recognized by a dimeric receptor composed of IFNAR1 and IFNAR2 subunits (blue, light blue subunits) [48,49,50]. After IFN binding and receptor dimerization, the juxtaposition of JAK1 (pink filled box) and TYK2 (orange filled box) increases kinase activity via transphosphorylation and subsequent STAT1 (light brown ellipse) and STAT2 (brown filled ellipse) and IRF9 (yellow filled ellipse) protein recruitment. STAT1/STAT2/IRF9 complex proteins are successively phosphorylated (blue circles), dimerized, and translocated to the nucleus, where ISG transcription is initiated after binding interferon-sensitive response element (ISRE) to produce mRNA for Interferon Alpha Inducible Protein 27 (IFI27; orange filled ellipse) [47]. Activation of the Transforming growth factor receptor is depicted by TGFB2 dimer binding to a tetrameric receptor structure composed of TGFBR1 (2 dark red subunits) and TGFBR2 (2 red subunits). Significant prognostic indicators that exhibited an increase in HR for OS outcomes are indicated by red outlines (TGFB2, IFNAR1, STAT1, IRF9, and IFI27 mRNAs). This figure was adapted from [51,52].

IFN-α is a crucial component of the initial response to invading infectious agents in a healthy immune system. It triggers the expression of numerous Interferon-stimulated genes (ISGs), including IFI27/ISG12a. IFI27 belongs to the IFI6/IFI27 family, which consists of a conserved 80 amino acid motif known as the ISG12 motif, found to be significantly stimulated by hepatitis B virus (HBV) infections [47]. Since IFN-I pathways also activate macrophages, we extended this observation to examine the mRNA expression of downstream processes’ IFNAR1 activation (Figure 8). Gene expression mRNA level analysis exhibited significant upregulation of mRNA levels in tumor tissue: IFNAR1 (3.4-fold increase, *p* < 0.0001), IRF9 (4.2-fold increase, *p* < 0.0001), STAT1 (7.1-fold increase, *p* < 0.0001), and IFI27 (66.3-fold increase, *p* < 0.0001). (Table S1).

Studies in other cancers have shown that the use of IFN-α, a ligand that activates IFNAR1, can promote stem-like properties in oral squamous cell carcinoma (OSCC) cells, whereby tumor xenografts treated with IFN-α increased the expression of stemness markers and tumor growth [53,54]. In vitro tests on OSCC cells treated with IFN-α have shown increased self-renewal capacity and stemness markers. Qadir et al. recently described a strong correlation between the death receptor CD95/Fas, IFN-I-dependent activation of STAT1, and stemness in various cancer types [55]. CD95 is an apoptosis-inducing death receptor but can also participate in various tumor-promoting activities. Chronic stimulation of CD95 in tumor cells has been reported to increase the number of cancer stem cells in breast cancer [56].

A previous study analyzed the levels of interferon regulatory factors (IRFs) 2, 6, 7, 8, and 9 in PDAC tumor and normal tissues. The results showed that the levels of these factors were significantly higher in tumors compared to normal tissues. In pancreatic cancer patients, the expression of IRF7 was significantly associated with the pathology stage. At the same time, high levels of IRF2, low levels of IRF3, and high levels of IRF6 were indicators of poorer overall survival. Furthermore, the study found that increased mRNA expression, amplification, and deep deletion were the most common types of genetic alterations of IRFs in PDAC. In addition, IRFs were positively correlated with the abundance of tumor-infiltrating immune cells, including B cells, CD8+ T cells, CD4+ T cells, macrophages, neutrophils, and dendritic cells in PC. The functional analysis indicated the involvement of IRFs in the T cell receptor signaling pathway, immune response, and Toll-like receptor signaling pathway. In conclusion, this study provides evidence supporting the involvement of IRFs in the progression of PDAC. These findings suggest that the expression of IRFs can serve as a potential biomarker for the diagnosis and prognosis of PDAC [57].

In addition to our study, multiple studies have shown that IFI27 is highly expressed in several cancers [58,59], such as ovarian cancer, hepatocellular carcinoma [60], and breast cancer [61]. Another study showed that overexpression of IFI27 increased the proliferation, migration, and invasion of Cholangiocarcinoma cells. Clinically, higher expression of IFI27 was associated with worse overall survival in patients with Cholangiocarcinoma [62]. A comprehensive bioinformatic analysis of the mRNA expression of IFI27 in PDAC revealed that the gene-encoding Interferon Alpha-Inducible IFI27 was significantly upregulated in pancreatic cancer tissues compared to normal tissues. Additionally, higher mRNA expression of IFI27 was found to be negatively correlated with the overall survival rate of pancreatic cancer patients. The functional annotation of IFI27 demonstrated its relationships to cellular immunity and metabolism, particularly glycolysis. Analysis of infiltrating immune cells showed that increased expression of IFI27 correlated with decreased CD8+ T cells and increased M2 macrophages [63].

Our findings supported the notion that interferon-alpha receptor activation and increased mRNA levels of ligands and receptors of the TGFB pathway play an essential role in the PDAC tumor microenvironment. This prompted the examination of the prognostic impact of TGFB2 mRNA in combination with components of the IFN-I pathway. To elucidate how high levels of TGFB2 and high levels of IRF9 (Figure 3) or IFI27 (Figure 4) impacted OS, we stratified patients into four groupings based on their expression levels of TGFB2 and IRF9 or IFI27 using the 50th percentile cut-offs for the range of mRNA expression levels of these genes. Patients with low levels of TGFB2 and IRF9 or IFI27 expression exhibited median OS times of 72 months. Patients with either high expression levels of TGFB2, IRF9, or IFI27 showed median OS times ranging from 16 to 20 months (*p* < 0.01 for all comparisons with the TGFB2^low^/IRF9 ^low^ or IFI27^low^). This suggested that knocking down TGFB2 and IRF9 or IFI27 expression results in a pronounced improvement in PDAC OS outcomes.

To test whether the effects of TGFB2, IRF9, or IFI27 mRNA were independent, we performed multivariate analyses of the hazard ratios of TGFB2 and IRF9 or IFI27 levels on OS were determined using the multivariate Cox proportional hazards model to adjust for age and TGFB2 statistical interaction with either IRF9 or IFI27 (Figure 5C,D). In this scenario, the beneficial effects of reduced TGFB2 levels were only observed in the context of low expression levels of IRF9 or IFI27, as demonstrated by the significant interaction term in the multivariate Cox proportional hazards models (Figure 5C,D). Our studies suggest that TGFB2 and IRF9 or IFI27 are overall independent prognostic markers for PDAC survival, but the impact of TGFB2 mRNA IRF9 or IFI27 is reduced at high IRF9/IFI27 mRNA levels. IFI27 is one of the most significantly upregulated genes in the PDAC TME (66.3-fold increase), with high levels of expression in tumor tissue (Mean ± SEM = 9.63 ± 0.12 log_2_ TPM), suggesting its potential to serve as a prognostic biomarker in these patients.

### 3.3. Targeting TGFB2 and IFN-I Response Gene Expression in PDAC Patients

To exploit targeting TGFB pathways in PDAC patients, TGF-β signaling inhibitors have been extensively researched in preclinical settings and have entered clinical development under five categories. The first category pertains to ligand inhibition, involving the delivery of antisense oligonucleotides (ASOs) either intravenously or through immune cell engineering to prevent TGF-β synthesis of AP12009, AP1104/AP15012, Lucanix™, and ISTH0036). The second category involves ligand traps and neutralizing antibodies that prevent TGF-β ligands from binding to receptors, including GC1008, 2G7, 1D11, LY2382770, and CAT-192. The third category pertains to a vaccine-based strategy. The fourth category involves small molecule inhibitors, which inhibit receptor kinase activity and prevent signal transduction, including SB431542, Ki 26894, SD208, LY2109761, IN-1130, LY2157299, TEW-7197, and PF-03446962. The fifth category employs intracellular peptide aptamers (and antagonists) (extensively reviewed in [64]). The anticancer compound OT-101 (Trabedersen) is a targeted antisense molecule that selectively binds to the human TGF-β2 mRNA (TGFB2). In a Phase I/II clinical trial, patients with PDAC treated with OT-101 and subsequent chemotherapy displayed significantly improved overall survival (OS) rates [65]. The mechanism of action of OT-101 involves the suppression of TGF-β signaling, which leads to the upregulation of cytokines, namely, IL-8, IL-15, IP-10, and HGF. A mixed analysis of the covariance model with OS as the covariate at each time point showed that anti-tumor cytokines IL-8 and IL-15 were significantly associated with OS during Cycle 1 of therapy [65]. These findings suggest that OT-101 can potentially improve the clinical outcomes of patients with PDAC and may serve as a valuable therapeutic agent in managing this disease. This led to the design of a clinical study to compare the efficacy and safety of OT-101 in combination with FOLFIRINOX (folinic acid, 5-FU, irinotecan, oxaliplatin) to FOLFIRINOX alone in patients with advanced and unresectable or metastatic pancreatic cancer (NCT06079346 submitted on 29 September 2023).

To identify inhibitors of the type-I interferon signaling pathway, a high-throughput screening study was conducted using the secreted embryonic alkaline phosphatase reporter gene assay against a library of 32,000 compounds that yielded 25 compounds of potential small molecule inhibitors. Subsequent characterization of these compounds was conducted to assess their cytotoxicity, effects on STAT phosphorylation, and activities in IFN regulatory factor (IRF) transcription [66]. Interestingly, a more recent study examining the role of enhancer of zeste homolog 2 (EZH2), which is an enzyme that adds methyl groups to the histone protein H3 at the lysine 27 position, leading to gene repression, showed that EZH2 expression is increased in the peripheral blood mononuclear cells and renal tissues of patients with systemic lupus erythematosus (SLE). This upregulation positively correlates with the overexpression of interferon-stimulated genes (ISGs). In vitro inhibition of EZH2 using either siRNAs or chemical inhibitors reduced the phosphorylation of STAT1 and the induction of ISGs stimulated by IFN-α (Wu et al., 2021) [67]. Our study’s findings provide for the use of an expanded set of pharmacological tools in combination therapies for PDAC patients, exploiting the knockdown of TGFB2 and components of the type-I interferon signaling pathway.

This present study suggests that abrogating TGFB2 mRNA levels and components of the IFN-α response (IFNAR1, STAT1, IRF9, and IFI27) can significantly improve OS outcomes in PDAC patients (Figure 5); to propose the use of inhibitors of IFNAR1 activation in conjunction with OT-101 would significantly improve OS outcomes in PDAC patients.

Patient biopsies could be obtained to measure the mRNA levels of TGFB2 and components of the IFN-I response pathway in a future clinical trial design. In cases where the biopsy sample demonstrated high levels of TGFB2 and the IFN-I pathway genes, the patient would be treated with both OT-101 and IFN-I pathway inhibitors. In the context of low IFN-pathway mRNA levels, treatment with OT-101 would be beneficial, or if the patient presented with low levels of TGFB2 mRNA, then treatment with IFN-I pathway inhibitor would potentially improve OS outcome.

### 3.4. High TGFB2 mRNA Expression Levels Exhibit Increases in Hazardratio (HR) Independent of 9 Prognostic Macrophage Markers (C1QA, CD74, HLA-DQB1, HLA-DRB1, HLA-F, IFI27, IRF9, LGALS9, and MARCO mRNA) in the TME, but the Impact of High TGFB2 mRNA Levels Is Reduced at High Levels of Macrophage Markers

A previous bioinformatic study reported a significant positive correlation of TGFB2 mRNA with TAM markers (CCl2, CD68, and IL10) and M2 markers (CD163, VSIG4, and MS4A4A) in PDAC patients’ mRNA levels [33]. We have also highlighted and extended the complex role of TGFB2 mRNA levels and markers for macrophages in the PDAC TME, such that TGFB2 mRNA impacts OS in PDAC patients independent of macrophages in the TME, as high TGFB2 levels showed shorter median OS times in patients with low macrophage abundance (Figure 2B). We further investigated the gene expression of TAM populations that impact OS independently of TGFB2 mRNA levels in the TME in PDAC patients by compiling a list of potential macrophage markers from published single-cell RNA-seq experiments and testing their impact and TGFB2 correlations to OS using a multivariate Cox proportional hazards model controlling for age and statistical interaction between TGFB2 and macrophage markers. In our survey of 81 macrophage markers, 54 exhibited significant increases in HR for TGFB2 independently of the macrophage markers (Table S2), thereby identifying potential markers for TAM populations contributing to the low abundance macrophage environment that exhibits the macrophage-independent impact of TGFB2 mRNA in PDAC patients (Figure 2). Of these, nine genes exhibited significant increases in HR for TGFB2 expression, macrophage mRNA marker expression, and a significant interaction term (C1QA, CD74, HLA-DQB1, HLA-DRB1, HLA-F, IFI27, IRF9, LGALS9, and MARCO). Gene expression profiles that compared upregulation of TGFB2 mRNA in tumor tissue revealed that TGFB2 was co-regulated with three (C1QA, HLA-F, and LGALS9) of these nine genes that also included mRNA expression levels of TGFB1, MRC1/CD206, TLR8, SPI1, C1QA, HLA-F, and LGALS9 in the cluster of genes (Figure 6B). The significant effect of the interaction term from the multivariate model suggested that macrophages expressing high levels of these nine genes significantly reduce the negative prognostic impact of high-TGFB2 mRNA. This effect is demonstrated by the four most significantly impacted increases in HR for the effect of high levels of TGFB2 mRNA presented in models for HLA-F, CD74, LGALS9, and IRF9, showing predicted OS profiles comparing high versus low expression of these four marker genes (see Figure 7). In normal tissues, MARCO was expressed at very low levels (<1 TPM) and significantly upregulated in tumor tissue (47-fold increase, *p* < 0.0001). MARCO was found to be co-regulated with IFI27 expression (Figure 6B) and exhibited a similar parameter profile for the multivariate Cox proportional hazards model (Figure 6A), suggesting that the detection of MARCO in tumor tissue would limit the impact of TGFB2 knockdown for OS improvements in PDAC patients. IFI27 is a highly expressed prognostic marker that was coregulated with MARCO, CD68, and APOC1 macrophage markers, and IFNAR1 was co-regulated with ADM, CD40, and IRF9, suggesting that IFN-I activation may operate via multiple subpopulations of TAMs in the PDAC TME.

A study that examined the single-cell RNA-seq method to characterize dynamic changes in TME of PDAC patients charted the emergence of four subsets of macrophages during malignancy, in which MARCO was co-expressed with SPP1 mRNA marker gene [19] pro-angiogenic TAMs in the development of PDAC [26], playing a role in epithelial–mesenchymal transition (EMT) and aiding in tumor invasion [21]. MARCO and CD163 mRNA expression in PDAC tissues is a negative prognostic marker for pancreatic cancer after surgery [68], and the application of anti-bodies against MARCO may present itself as an attractive therapeutic approach to remodel the TME towards susceptibility to immunotherapies [69].

Single-cell transcriptomic profiles obtained from resected PDAC primary tumors and matched liver metastases identified a subset of TAMs expressing HLA-DRA, CD74, C1QA mRNA [20] that also exhibited independent prognostic effects with significant interaction terms with TGFB2 mRNA expression in the multivariate models reported in our study (Figure 6A), thereby suggesting cooperation between TGFB2 and this subtype of TAMs in the metastasis of pancreatic cancer cells. Future therapies would require either targeting TGFB2 mRNA expression and inhibiting this subtype of TAM, or targeting mRNA expression of HLA-DRA, CD74, or C1QA.

Examining the upregulation of gene expression in tumors relative to normal tissue (Figure 6A), it was found that LGALS9 was co-regulated with TGFB2 mRNA levels. The multivariate Cox proportional hazards model that included statistical interaction of TGFB2 and LGALS9 showed that TGFB2 exhibited one of the four most impactful increases in HR. However, the effect was abolished at high levels of LGALS9 (Figure 7F). This prediction model suggests that either LGALS9 can be used as a biomarker to identify patients that would not respond to TGFB2 mRNA blockade or inhibition of LGALS9 in combination with TGFB2 abrogation to achieve survival benefit for PDAC patients. A study that applied a computational approach to characterize ligand-receptor interactions between macrophages and tumor ductal cells using the CellphoneDB algorithm to interrogate single-cell transcriptomics data showed significant LGALS9-CD44 and LGALS9-MET interactions were correlated with worse overall survival in PDAC TME, and LGALS9 was over-expressed in the macrophage subset [18]. LGALS9 mRNA, upregulated 16.5-fold in tumor tissue, produces Galectin-9 protein that interacts with tumors independently to influence tumor progression, functioning as an immune checkpoint and merging as a target for immunotherapy [70].

TGFB2 was found in our analysis to be a negative prognostic indicator in low macrophage and low neoantigen TME PDAC tumors (Figure 2C), which was also reflected in the multivariate analysis of OS outcomes identifying TGFB2 with shorter OS times at low levels of Major Histocompatibility Complex, Class I, F, HLA-F (Figure 7). Similar model parameter profiles were observed for Major Histocompatibility Complex, Class II, DQ Beta 1 (HLA-DQB1) and Major Histocompatibility Complex, Class II, DR Beta 1 (HLA-DRB1) mRNA gene expression profiles suggesting that under conditions of low neoantigens or low expression of MHC class I or II mRNA that codes for cell surface receptors presenting antigens to T-cells, TGFB2 mRNA expression results in worse OS outcomes (Figure 6). Examining mRNA expression levels in tumors versus normal comparisons (Figure 6B) showed that CD74, a type-II transmembrane glycoprotein which has been shown to function as a chaperone in transporting MHC II molecules involved in antigen presentation [71], was coregulated with HLA-DQB1, HLA-DRB1, and HLA-DRA, and served as a marker for M1-like macrophage infiltration in 32 cancers including PDAC from the TCGA dataset [72]. HLA-DRA (9.3 ± 0.1; 31-fold increase) and CD74 (Mean ± SEM = 10.94 ± 0.08; 21.8-fold) exhibited the most significant increase in expression levels in tumor tissue relative to normal tissue, suggesting its potential use as a biomarker for PDAC prognosis, whereby PDAC patients with low levels of CD74 or HLA-DRA would benefit from TGFB2 mRNA knockdown.

Our research shows that targeting the tumor microenvironment requires a careful analysis of multiple dependencies among interacting biochemical components in TAMs present in the TME. Our findings show that inhibiting TGFB2 mRNA levels to improve overall survival (OS) is effective only when the mRNA expression levels of the IFNAR1/STAT1/IRF9/IFI27 axis are low. This improvement in OS was observed for TGFB2 independent of TGFB1 and TGFB3 ligands. Indeed, improving treatment outcomes is proving to be challenging, considering recent disappointing reports from clinical trials targeting the TGFB pathway that did not specifically block the mRNA production of TGFB2. Trials employing bintrafusp alfa, a first-in-class bifunctional fusion protein designed to block TGF-β and PD-L1, were evaluated in two randomized phase II studies in lung cancer and one in biliary tract cancer. However, in these studies (NCT04727541, NCT04066491), bintrafusp alfa did not demonstrate superiority over standard-of-care therapies [73,74], and demonstrated very modest improvements in metastatic nasopharyngeal carcinoma [75]. Very recently, Novartis discontinued the development of the pan- anti-TGFß monoclonal antibody NIS793 for the treatment of patients with pancreatic cancer and other malignancies (NCT04390763, NCT04935359, NCT05417386). We propose that TGFB2 mRNA needs to be specifically targeted for treatment. This targeting is necessary but insufficient, because the activation of IFN-I and subpopulations of TAMs also contributes to clinical outcomes independent of TGFB2 mRNA levels. Furthermore, the multivariate Cox proportional hazards models suggested that the impact of TGFB2 mRNA is reduced at high levels of IRF9/IFI27 or markers for TAM subpopulations.

The present study has significant limitations, as the bioinformatics-based analyses were used without additional supportive laboratory testing of TGFB2 mRNA levels and mRNA levels of TAM markers validated using platforms such as quantitative RT-PCR and immunohistochemistry tests on specific cellular components. Our findings of mRNA expression levels correlated with survival outcomes will require further experimental validation from PDAC tumor biopsies in future clinical trials monitoring TGFB2 mRNA, IFN-I pathway, and TAM markers for protein, and mRNA measured using quantitative reverse transcription polymerase chain reaction methods. These considerations will enable a more nuanced assessment of the impact of tumor heterogeneity characterized by TAM component and TGFB2/IFN-I activation mechanisms to predict clinical outcomes and to provide biomarkers for stratified analyses based on molecular characteristics of PDAC tumors.

## 4. Materials and Methods

### 4.1. Overall Survival (OS) Outcomes for PDAC Patients Were Stratified According to Expression Levels of TGFB2 and Target Genes in the TME

We utilized an integrated database portal [76] for PDAC for Kaplan–Meier analysis to determine the prognostic impact of TGFB ligand (TGFB1, TGFB2, TGFB3 median expression cut-off values for high versus low patient sub-groupings) on OS for all PDAC patients (N = 177), PDAC patients with low macrophage populations (N = 68), and PDAC patients with low macrophage and low neoantigen levels (N = 47) (http://kmplot.com/analysis/index.php?p=service&cancer=pancancer_rnaseq, accessed on 23 May 2024).

We further analyzed patient-level clinical metadata and RNA sequencing-based batch normalized mRNA expression data for 177 patients diagnosed with PDAC using the cBioportal repository (https://www.cbioportal.org/study/summary?id=paad_tcga_pan_can_atlas_2018, accessed on 13 August 2022). Data files harboring RSEM-calculated TPM expression values were correlated to OS outcomes by compiling patient-level data directly downloaded from the cBioportal as a .tsv file. To calculate the percentiles of gene expression from 177 PDAC patients, log_2_ transformed TPM values were stratified according to medians of ranked values. Four patient groups were then formed based on their expression levels of TGFB2 and Gene2 (Macrophage marker genes expressed in the tumor microenvironment that also included TGFB1, TGFB2, TGFB3, TGFBR1, TGFBR2, TGFBR3, IFNAR1, STAT1, IRF9, and IFI27): high expression of both (TGFB2^high^/Gene2^high^; higher than or equal to the 50th percentile of both TGFB2 and Gene2); low expression of both (TGFB2^low^/Gene2^low^; lower than the 50th percentile of both TGFB2 and Gene2); and combinations of high and low expression levels for both genes under investigation (TGFB2^high^/Gene2^low^, and TGFB2^low^/Gene2^high^). OS curves censored at 120 months were then compared between these groups to assess the survival impact of the combinations of TGFB2 and Gene2 levels. We also evaluated the impact of either TGFB2^high^/Gene2^high^ versus the remaining patients on OS to test the effect of the subset of patients with high mRNA gene expression levels. We analyzed the patient subsets’ OS outcomes using the Kaplan–Meier (KM) method tested for statistical significance using the log-rank chi-square test, implemented utilizing R-based software packages (https://cran.r-project.org/ accessed 9 November 2021) including survival_3.2-13, survminer_0.4.9, and survMisc_0.5.5. To present the treatment outcomes in a graphical format, we plotted graphs using dplyr_1.0.7, ggplot2_3.3.5, and ggthemes_4.2.4 implemented in R. We considered *p*-values less than 0.05 significant after adjusting for multiple comparisons across four groups (6 comparisons) using the Benjamini and Hochberg method.

### 4.2. Hazard Ratio Comparisons for PDAC Patients Are Used to Determine the Independent Effect of Low TGFB2 mRNA Levels and Target Gene Control on Target Gene Expression Levels, Age, and Interaction between TGFB2 and the Target Gene

We implemented univariate Cox proportional hazards models to assess the prognostic impact of TGFB ligands (TGFB1/2/3; N = 177), receptors (TGFBR1/2; N = 177), and age (N = 178) on the overall survival (OS) of PDAC patients. Variables that indicated significant effects on OS outcomes were further investigated in a multivariate model that included age as a confounding variable.

In addition, we investigated the prognostic impact on TGFB2 mRNA expression levels of markers for Gene2 by performing Multivariate analyses utilizing the Cox proportional hazards model to assess the individual effects of TGFB2 and Gene2 mRNA levels on OS. This analysis controlled for age at diagnosis and the interaction between TGFB2 and Gene2. Briefly, the model included (i) The mRNA expression level for TGFB2 as a categorical variable comparing high versus low TGFB2 mRNA expression levels at 50% cut-off for expression values, (ii) The mRNA expression level for Gene2 as a categorical variable comparing high versus low Gene2 mRNA expression levels (50% cut-off), (iii) age at diagnosis, and (iv) interaction term implemented in R (survival_3.2-13 ran in R version 4.1.2). Forest Plots were utilized to visualize the Hazard ratios for Cox proportional hazards models for OS outcomes (survminer_0.4.9 ran in R version 4.1.2 (1 November 2021)). The life table hazard ratios (HRs) were estimated using the exponentiated regression coefficient for Cox proportional hazards analyses implemented in R (survival_3.2-13 ran in R version 4.1.2). We analyzed the effect of adding an interaction term (TGFB2 x Gene2) as the fourth parameter in the Cox proportional hazards model. This enabled the determination of the independent impact of TGFB2 and Gene2 in models with the interaction term. To better understand how different combinations of TGFB2 high and low mRNA expression groups in the context of Gene2 high and low mRNA expression groups affect predicted survival proportion at any given time, we plotted and calculated the shift in the baseline OS curve for 177 PDAC patients using parameters from the interaction model. We compared the median OS times for patients with high and low TGFB2 expression levels who expressed high or low levels of Gene2. A significant interaction effect from the interaction model would indicate differences in OS times for patients with high versus low TGFB2 expression levels depending on their Gene2 levels. Interaction parameters with HR less than 1 suggested that the negative prognostic impact of high TGFB2 mRNA levels is enhanced at low levels of Gene2 expression.

### 4.3. TGFB2 mRNA Expression Correlation with the mRNA Levels of Macrophage Prognostic Markers in the TME

A curated list of 81 macrophage mRNA markers, identified in classically activated M1 (CAM) or alternatively activated M2 (AAM) subtypes [77,78,79,80]. Additionally, markers were obtained from single-cell RNA sequencing experiments that utilized PDAC tumor tissues [16,17,18,19,20] for investigations in this study. Multivariate analyses using the Cox proportional hazards model examined the independent impact of TGFB2 and Gene2 on OS. To control for age at diagnosis and interaction effect, we screened 81 models (TGFB2 interaction with TAM markers, TGFB genes, Interferon receptors/STAT1/IRF9/IFI27) using multivariate Cox models (Table S2). Of these, 54 exhibited significant increases in HR for TGFB2 independently of Gene2 markers. To identify prognostically relevant markers, we further examined TGFB2-Gene pairs that exhibited either significant increases in both TGFB2 and Gene2 OS hazard ratios or an increase in TGFB2 hazard ratio and a significant (*p* < 0.05) interaction effect revealing 21 gene markers that impact OS in combination with TGFB2 levels (ADM, APOC1, C1QA, CCL18, CCL5, CD40, CD74, EGLN3, HHLA2, HLA-DQB1, HLA-DRA, HLA-DRB1, HLA-F, IFI27, IFNAR1, IRF9, LGALS9, MARCO, SPI1, TGFB1, and TLR8 mRNAs). A total of 17 exhibited significant increases in HR for TGFB2 expression and the interaction term with Gene2 (APOC1, C1QA, CCL18, CCL5, CD40, CD74, HLA-DQB1, HLA-DRA, HLA-DRB1, HLA-F, IFI27, IRF9, LGALS9, MARCO, SPI1, TGFB1, and TLR8), and 13 showed significant increases in HR for TGFB2 and Gene2 (ADM, C1QA, CD74, EGLN3, HHLA2, HLA-DQB1, HLA-DRB1, HLA-F, IFI27, IFNAR1, IRF9, LGALS9, and MARCO). Nine genes exhibited significant increases in HR for TGFB2 expression, Gene2 expression, and a significant interaction term (C1QA, CD74, HLA-DQB1, HLA-DRB1, HLA-F, IFI27, IRF9, LGALS9, and MARCO mRNAs).

### 4.4. Differential Expression of mRNA Comparing PDAC Tumors Versus Normal Pancreatic Tissue Samples

We utilized log2 transformed transcripts per million (TPM) summarized RNAseq data files (https://toil-xena-hub.s3.us-east-1.amazonaws.com/download/TcgaTargetGtex_rsem_gene_tpm.gz; Full metadata accessed on 25 July 2023) downloaded from the UCSC Xena web platform (https://xenabrowser.net/datapages/, accessed on 25 July 2023) to compare gene expression levels for 178 tumor tissue samples (search term: “TCGA Pancreatic Adenocarcinoma”) versus 167 pancreatic tissue samples (search term: “GTEX Pancreas”). This resource reports results from the UCSC Toil RNAseq recompute compendium, which is a standardized realigned and recalculated gene and transcript expression dataset for all TCGA, and GTEx that enables users to contrast gene and transcript expression between TCGA “tumor” samples and corresponding GTEx “normal” samples [81]. We applied a two-way ANOVA model to identify differentially expressed genes to compare normal versus tumor tissue samples. The log_2_ transformed TPM values for Gene and Tissue were included as fixed factors, along with one interaction term to investigate gene-level effects for normal and tumor tissues (Gene x Tissue). For each gene, we conducted a comparison between normal and tumor samples blocked by the Gene factor and then determined significance by adjusting the *p*-value using the false discovery rate algorithm provided for in the R-package (FDR corrected for all pairs) calculations performed in R using multcomp_1.4-17 and emmeans_1.7.0 packages ran in R version 4.1.2 (1 November 2021) with RStudio front end (RStudio 2021.09.0+351 “Ghost Orchid” Release). Bar chart graphics were constructed using the ggplot2_3.3.5 R package.

We used a two-way hierarchical clustering technique to organize expression patterns such that sample and Gene expression displaying similar mRNA expression profiles were grouped together using the average distance metric (default Euclidean distance implemented using the heatmap.2 function in the R package gplots_3.1.1). The cluster figure displayed the mean expression levels in tumor tissue centered to the normal pancreas expression levels representing log_2_-transformed fold-change values. The associated dendrograms organized and depicted expression levels of co-regulated genes for both (rows) and pancreatic cancer patients (columns).

## 5. Conclusions

Pancreatic adenocarcinoma tumors exhibit increased levels of mRNA expression for components of the Transforming growth factor-β pathway (TGFB1/2/3, TGFBR1/2/3) and Interferon Type I pathways (IFNAR1, STAT1, IRF9 and IFI27). These increased levels of mRNA expression were found to be prognostically significant, whereby patients with either high expression levels of TGFB2, IRF9, or IFI27 showed median OS times ranging from 16 to 20 months (*p* < 0.01 compared to 72 months for patients with low levels of expression for both TGFB2 and either IRF9 or IFI27). This suggested that knocking down TGFB2 and IRF9 or IFI27 mRNA expression results in a pronounced improvement in OS outcomes. Furthermore, increases in hazard ratios were independent for high levels of TGFB2 and IRF9 or IFI27, controlling for age and interaction effects using a multivariate Cox proportional hazards model. Examination of TAM markers applying multivariate Cox proportional hazards models revealed nine genes exhibiting significant increases in the hazard ratio for TGFB2 expression, TAM marker gene expression, and a significant interaction term between TGFB2 and marker mRNA expression (C1QA, CD74, HLA-DQB1, HLA-DRB1, HLA-F, IFI27, IRF9, LGALS9, and MARCO), suggesting that the impact on OS for TGFB2^high^ group of PDAC patients is modified by a subpopulation of TAM expressing these marker genes, whereby the negative prognostic impact of TGFB2 mRNA levels is observed at low, not high, TAM marker mRNA levels. Of these genes, the highest levels of expression in tumor tissue were observed for CD74 (Mean ± SEM = 10.94 ± 0.08; 21.8-fold increase relative normal tissue), IFI27 (9.63 ± 0.12; 66.3-fold increase), and HLA-DRA (9.3 ± 0.1; 31-fold increase), and therefore can potentially be used as biomarkers for PDAC prognosis. Highly significant impacts of PDAC OS were observed with respect to prognostic impacts of TGFB2, LGALS9 mRNA, and the interaction term for the Cox multivariate regression models, suggesting that Gal-9 immune checkpoint protein could be targeted for therapy in combination with TGFB2 blockade with molecules such as OT-101. Results of our research suggest that a wider range of drugs will be required to be used in combination treatments for patients with pancreatic adenocarcinoma. Targeting both TGFB2, macrophage polarization in the TME, and elements of the type-I interferon signaling pathway could greatly enhance survival rates for these patients.

## Data Availability

We utilized log2 transformed transcripts per million (TPM) summarized RNAseq data files (https://toil-xena-hub.s3.us-east-1.amazonaws.com/download/TcgaTargetGtex_rsem_gene_tpm.gz; Full metadata) downloaded from the UCSC Xena web platform (https://xenabrowser.net/datapages/, accessed on 25 July 2023). We analyzed clinical metadata and RNA sequencing-based mRNA expression data for 177 patients diagnosed with PDAC (https://www.cbioportal.org/study/summary?id=paad_tcga_pan_can_atlas_2018, accessed on 13 August 2022).

## Supplementary Materials

The following supporting information can be downloaded at: https://www.mdpi.com/article/10.3390/ijms252011221/s1.
