# Supplementary material for: TGFB2 mRNA Levels Prognostically Interact with Interferon-Alpha Receptor Activation of IRF9 and IFI27, and an Immune Checkpoint LGALS9 to Impact Overall Survival in Pancreatic Ductal Adenocarcinoma
Source: Int J Mol Sci. 2024 Oct 18;25(20):11221. doi: 10.3390/ijms252011221 (PMC11508538; doi:10.3390/ijms252011221)
Supplement: Supplementary file 1 [file ijms-25-11221-s001.zip › ijms-3197363-supplementary.pdf]

Table S1. Comparing the mRNA expression levels in normal pancreatic tissue and tumor samples obtained from patients diagnosed with PDAC.

	Normal Tissue (N=167)		PDAC Tumor Tissue (N=178)			
Gene	Mean ± SEM	Median (Range)	Mean ± SEM	Median (Range)	Fold Change	P-value
IFI27	3.58 ± 0.13	3.66 (-9.97 - 8.36)	9.63 ± 0.12	9.78 (4.53 - 12.46)	66.3	<0.0001
IFNAR1	2.18 ± 0.11	2.35 (-9.97 - 3.68)	3.94 ± 0.04	3.94 (1.72 - 5.03)	3.4	<0.0001
IRF9	4.27 ± 0.14	4.38 (-9.97 - 7.12)	6.33 ± 0.06	6.39 (2.97 - 7.6)	4.2	<0.0001
STAT1	2.9 ± 0.12	3 (-9.97 - 6.32)	5.73 ± 0.07	5.74 (1.94 - 8.13)	7.1	<0.0001
TGFB1	2.38 ± 0.13	2.4 (-9.97 - 5.83)	5.42 ± 0.06	5.65 (1.82 - 7.29)	8.3	<0.0001
TGFB2	-0.22 ± 0.12	-0.09 (-9.97 - 3.21)	2.76 ± 0.11	2.82 (-3.05 - 6.54)	7.9	<0.0001
TGFB3	3.83 ± 0.14	3.79 (-9.97 - 7.23)	4.85 ± 0.11	4.98 (-2.11 - 8.21)	2.0	<0.0001
TGFBR1	1.26 ± 0.11	1.32 (-9.97 - 4.51)	4.02 ± 0.07	4.15 (0.33 - 5.57)	6.8	<0.0001
TGFBR2	3.77 ± 0.13	3.91 (-9.97 - 6.78)	5.74 ± 0.07	5.85 (2.02 - 7.49)	3.9	<0.0001
TGFBR3	1.99 ± 0.12	2.06 (-9.97 - 4.69)	2.34 ± 0.09	2.37 (-1.35 - 5.7)	1.3	0.021

Table S2. Multivariate Cox proportional hazards models impacting OS in PDAC patients. Hazard ratios were calculated for macrophage markers, TGFB ligands and receptors, interferon alpha receptors, and interferon alpha response genes (STAT1, IRF9, and IFI27).

	TGFB2		Gene 2		Age at Diagnosis		TGFB2 x Gene 2	
Gene 2	HR (95% CI)	P-value	HR (95% CI)	P-value	HR (95% CI)	P-value	HR (95% CI)	P-value
CD74	3.98 (2.12-7.44)	0	2.33 (1.24-4.38)	0.009	1.03 (1.01-1.05)	0.009	0.19 (0.08-0.44)	0
HLA-F	4.65 (2.21-9.78)	0	3.41 (1.64-7.07)	0.001	1.03 (1.01-1.05)	0.006	0.18 (0.07-0.45)	0
IRF9	3.32 (1.71-6.47)	0	3.5 (1.82-6.72)	0	1.03 (1.01-1.05)	0.014	0.27 (0.11-0.62)	0.002
LGALS9	3.55 (1.79-7.06)	0	2.41 (1.24-4.7)	0.009	1.03 (1.01-1.06)	0.003	0.27 (0.11-0.65)	0.003
APOC1	2.91 (1.59-5.35)	0.001	1.5 (0.81-2.8)	0.199	1.03 (1.01-1.05)	0.002	0.36 (0.15-0.82)	0.015
CCL5	2.87 (1.58-5.21)	0.001	1.58 (0.84-2.95)	0.153	1.03 (1.01-1.05)	0.005	0.35 (0.15-0.81)	0.014
CD40	2.79 (1.5-5.2)	0.001	1.59 (0.84-2.98)	0.151	1.03 (1.01-1.05)	0.003	0.39 (0.17-0.91)	0.029
HLA-DRA	2.86 (1.55-5.28)	0.001	1.79 (0.96-3.34)	0.068	1.03 (1.01-1.05)	0.006	0.35 (0.15-0.82)	0.015
MARCO	3.13 (1.64-5.97)	0.001	2.4 (1.28-4.51)	0.006	1.03 (1.01-1.05)	0.005	0.3 (0.13-0.69)	0.005
SPI1	2.75 (1.54-4.91)	0.001	1.22 (0.65-2.29)	0.533	1.03 (1.01-1.05)	0.002	0.39 (0.17-0.91)	0.03
TGFB1	2.73 (1.52-4.9)	0.001	1.49 (0.78-2.84)	0.23	1.03 (1.01-1.05)	0.005	0.38 (0.16-0.89)	0.027
TLR8	2.82 (1.54-5.18)	0.001	1.71 (0.92-3.17)	0.091	1.03 (1.01-1.05)	0.003	0.36 (0.16-0.84)	0.017
CCL18	2.75 (1.47-5.14)	0.002	1.7 (0.92-3.16)	0.092	1.03 (1.01-1.05)	0.005	0.39 (0.17-0.9)	0.028
CTRB2	2.61 (1.43-4.75)	0.002	1.24 (0.66-2.35)	0.506	1.03 (1.01-1.05)	0.003	0.47 (0.2-1.09)	0.079
HLA-DQB1	2.71 (1.44-5.12)	0.002	1.97 (1.04-3.71)	0.037	1.03 (1.01-1.05)	0.004	0.41 (0.17-0.94)	0.036
HLA-DRB1	2.82 (1.45-5.47)	0.002	2.21 (1.16-4.22)	0.016	1.03 (1.01-1.05)	0.008	0.4 (0.17-0.93)	0.034
ISG15	2.56 (1.41-4.65)	0.002	1.38 (0.74-2.56)	0.307	1.03 (1.01-1.05)	0.005	0.44 (0.19-1.01)	0.054
C1QA	2.57 (1.37-4.84)	0.003	1.95 (1.05-3.63)	0.036	1.03 (1.01-1.05)	0.006	0.42 (0.18-0.96)	0.04
CCR2	2.44 (1.37-4.35)	0.003	1.16 (0.62-2.18)	0.638	1.03 (1.01-1.05)	0.004	0.5 (0.22-1.15)	0.104
IFI27	2.74 (1.42-5.28)	0.003	2.56 (1.34-4.87)	0.004	1.02 (1-1.05)	0.019	0.4 (0.17-0.92)	0.032
CLPS	2.34 (1.3-4.21)	0.004	1.35 (0.72-2.53)	0.343	1.03 (1.01-1.05)	0.006	0.52 (0.22-1.19)	0.12
CTRB1	2.44 (1.34-4.45)	0.004	1.31 (0.7-2.46)	0.4	1.03 (1.01-1.05)	0.004	0.5 (0.21-1.16)	0.108
MRC1	2.53 (1.35-4.73)	0.004	1.87 (1-3.51)	0.051	1.03 (1.01-1.05)	0.003	0.44 (0.19-1.02)	0.055
CD68	2.42 (1.31-4.48)	0.005	1.41 (0.76-2.62)	0.277	1.03 (1.01-1.05)	0.004	0.5 (0.22-1.15)	0.103
HLA-DQA1	2.4 (1.31-4.41)	0.005	1.64 (0.87-3.06)	0.123	1.03 (1.01-1.05)	0.007	0.49 (0.21-1.12)	0.091
GRN	2.32 (1.28-4.22)	0.006	0.94 (0.51-1.75)	0.85	1.03 (1.01-1.06)	0.001	0.5 (0.22-1.17)	0.112
IFNAR2	2.39 (1.28-4.46)	0.006	1.66 (0.89-3.09)	0.112	1.03 (1.01-1.05)	0.005	0.51 (0.22-1.18)	0.117
MT1G	2.44 (1.29-4.62)	0.006	1.85 (0.97-3.51)	0.061	1.03 (1.01-1.05)	0.013	0.5 (0.21-1.16)	0.108
CCL7	2.44 (1.28-4.63)	0.007	1.73 (0.92-3.26)	0.088	1.03 (1.01-1.05)	0.008	0.5 (0.22-1.16)	0.107
CD163	2.36 (1.27-4.38)	0.007	1.67 (0.9-3.09)	0.106	1.03 (1.01-1.05)	0.004	0.5 (0.22-1.15)	0.104
FCGR3A	2.41 (1.27-4.58)	0.007	1.74 (0.94-3.24)	0.079	1.03 (1.01-1.05)	0.005	0.47 (0.2-1.1)	0.083
LYZ	2.31 (1.24-4.28)	0.008	1.16 (0.61-2.2)	0.652	1.03 (1.01-1.05)	0.005	0.48 (0.2-1.15)	0.099
NUPR1	2.28 (1.22-4.24)	0.009	1.72 (0.92-3.21)	0.09	1.03 (1.01-1.05)	0.005	0.56 (0.24-1.28)	0.17
AIF1	2.26 (1.21-4.22)	0.01	1.43 (0.77-2.68)	0.259	1.03 (1.01-1.05)	0.005	0.55 (0.24-1.29)	0.171
FGFR2	2.23 (1.21-4.11)	0.01	1.31 (0.7-2.44)	0.403	1.03 (1.01-1.05)	0.009	0.58 (0.25-1.34)	0.204
CD86	2.19 (1.2-4.01)	0.011	1.51 (0.81-2.83)	0.194	1.03 (1.01-1.05)	0.005	0.56 (0.24-1.3)	0.178
MSR1	2.21 (1.2-4.06)	0.011	1.2 (0.64-2.25)	0.568	1.03 (1.01-1.05)	0.004	0.61 (0.26-1.42)	0.252
TGFBR3	2.16 (1.2-3.89)	0.011	1.05 (0.56-1.95)	0.877	1.03 (1.01-1.05)	0.004	0.62 (0.27-1.43)	0.263
CXCL3	2.21 (1.17-4.16)	0.014	1.32 (0.7-2.5)	0.392	1.03 (1.01-1.05)	0.006	0.59 (0.25-1.39)	0.226
IFNAR1	2.27 (1.18-4.36)	0.014	2.22 (1.17-4.22)	0.015	1.03 (1.01-1.05)	0.004	0.51 (0.22-1.2)	0.122

TGFB3	2.1 (1.16-3.8)	0.015	1.03 (0.55-1.93)	0.931	1.03 (1.01-1.05)	0.004	0.69 (0.3-1.59)	0.381
ADM	2.21 (1.16-4.21)	0.016	1.98 (1.06-3.73)	0.033	1.03 (1.01-1.05)	0.004	0.58 (0.25-1.34)	0.202
TGFBR2	2.09 (1.14-3.83)	0.017	1.37 (0.72-2.6)	0.333	1.03 (1.01-1.05)	0.005	0.62 (0.26-1.45)	0.269
FCN1	2.06 (1.13-3.75)	0.018	1.35 (0.73-2.5)	0.344	1.03 (1.01-1.05)	0.005	0.65 (0.29-1.5)	0.314
HHLA2	2.2 (1.15-4.24)	0.018	2.1 (1.1-4)	0.025	1.03 (1.01-1.05)	0.006	0.59 (0.25-1.38)	0.226
FCER1G	2.08 (1.12-3.89)	0.021	1.65 (0.89-3.08)	0.114	1.03 (1.01-1.05)	0.007	0.62 (0.27-1.42)	0.256
CLEC10A	1.95 (1.1-3.45)	0.022	0.83 (0.44-1.58)	0.574	1.03 (1.01-1.05)	0.006	0.83 (0.35-1.92)	0.657
EGLN3	2.11 (1.1-4.03)	0.024	1.9 (1.01-3.57)	0.046	1.03 (1.01-1.05)	0.005	0.63 (0.27-1.47)	0.286
RGS1	2.06 (1.1-3.85)	0.024	1.6 (0.85-2.98)	0.143	1.03 (1.01-1.05)	0.007	0.67 (0.29-1.55)	0.347
CCL4	1.97 (1.09-3.56)	0.025	1 (0.54-1.86)	0.987	1.03 (1.01-1.05)	0.004	0.75 (0.32-1.72)	0.494
TLR1	1.97 (1.07-3.61)	0.028	1.19 (0.64-2.23)	0.582	1.03 (1.01-1.05)	0.005	0.73 (0.32-1.69)	0.463
MERTK	1.84 (1.04-3.28)	0.038	0.85 (0.45-1.61)	0.621	1.03 (1.01-1.05)	0.008	0.9 (0.38-2.1)	0.804
FCGBP	1.93 (1.02-3.65)	0.044	1.4 (0.75-2.62)	0.287	1.03 (1.01-1.05)	0.01	0.75 (0.32-1.73)	0.497
SLC16A3	1.88 (1.02-3.48)	0.044	1.49 (0.8-2.78)	0.212	1.03 (1.01-1.05)	0.006	0.75 (0.33-1.74)	0.51
TOP2A	2.03 (1-4.14)	0.051	2.48 (1.28-4.79)	0.007	1.03 (1.01-1.05)	0.005	0.77 (0.32-1.84)	0.556
VEGFA	1.77 (0.98-3.18)	0.057	0.99 (0.53-1.84)	0.977	1.03 (1.01-1.05)	0.005	0.9 (0.39-2.06)	0.804
HBEGF	1.8 (0.97-3.34)	0.06	1.5 (0.81-2.8)	0.198	1.03 (1.01-1.05)	0.005	0.89 (0.38-2.04)	0.777
STAT1	1.84 (0.97-3.46)	0.06	2 (1.08-3.71)	0.029	1.03 (1.01-1.05)	0.005	0.7 (0.3-1.63)	0.413
TGFBR1	1.81 (0.96-3.42)	0.065	1.64 (0.88-3.04)	0.117	1.03 (1.01-1.05)	0.003	0.74 (0.32-1.72)	0.485
CX3CL1	1.76 (0.95-3.26)	0.073	1.18 (0.63-2.22)	0.61	1.03 (1.01-1.05)	0.005	0.87 (0.37-2.05)	0.752
CD80	1.74 (0.94-3.22)	0.076	1.22 (0.65-2.28)	0.537	1.03 (1.01-1.05)	0.005	0.89 (0.38-2.07)	0.782
PLAUR	1.68 (0.92-3.07)	0.091	0.98 (0.52-1.84)	0.953	1.03 (1.01-1.05)	0.005	1.01 (0.43-2.35)	0.983
FCER1A	1.59 (0.9-2.83)	0.113	0.71 (0.37-1.37)	0.309	1.03 (1.01-1.05)	0.007	1.23 (0.53-2.9)	0.628
TLR2	1.62 (0.89-2.95)	0.116	0.84 (0.45-1.57)	0.583	1.03 (1.01-1.05)	0.006	1.13 (0.48-2.64)	0.78
GALC	1.58 (0.89-2.81)	0.122	0.62 (0.33-1.17)	0.143	1.03 (1.01-1.05)	0.007	1.23 (0.53-2.83)	0.632
CCL3	1.59 (0.87-2.89)	0.129	0.85 (0.46-1.57)	0.607	1.03 (1.01-1.05)	0.005	1.12 (0.49-2.57)	0.784
COPA	1.63 (0.86-3.09)	0.134	1.17 (0.63-2.17)	0.627	1.03 (1.01-1.05)	0.006	1.06 (0.46-2.43)	0.891
HLA-G	1.63 (0.86-3.11)	0.135	1.49 (0.79-2.79)	0.213	1.03 (1.01-1.05)	0.003	1.18 (0.51-2.74)	0.693
CD14	1.51 (0.83-2.77)	0.177	0.83 (0.44-1.55)	0.559	1.03 (1.01-1.05)	0.005	1.25 (0.54-2.89)	0.598
CCL2	1.48 (0.84-2.63)	0.179	0.74 (0.4-1.38)	0.339	1.03 (1.01-1.05)	0.006	1.34 (0.58-3.08)	0.495
CSF1	1.47 (0.81-2.69)	0.205	0.73 (0.37-1.44)	0.366	1.03 (1.01-1.05)	0.005	1.43 (0.59-3.48)	0.428
ICAM1	1.48 (0.8-2.72)	0.207	1.05 (0.56-1.97)	0.875	1.03 (1.01-1.05)	0.004	1.23 (0.53-2.87)	0.625
STMN1	1.49 (0.8-2.77)	0.212	1.27 (0.68-2.37)	0.45	1.03 (1.01-1.05)	0.003	1.2 (0.52-2.77)	0.675
TLR4	1.47 (0.8-2.72)	0.214	0.89 (0.47-1.66)	0.707	1.03 (1.01-1.05)	0.006	1.29 (0.55-3)	0.555
S100A9	1.47 (0.8-2.7)	0.22	1.15 (0.62-2.15)	0.661	1.03 (1.01-1.05)	0.008	1.23 (0.53-2.85)	0.635
TEK	1.43 (0.8-2.55)	0.232	0.7 (0.36-1.34)	0.278	1.03 (1.01-1.05)	0.009	1.48 (0.63-3.51)	0.368
KDR	1.35 (0.76-2.41)	0.303	0.54 (0.27-1.04)	0.067	1.03 (1.01-1.05)	0.014	1.79 (0.74-4.32)	0.193
MAFB	1.34 (0.77-2.34)	0.305	0.49 (0.26-0.94)	0.031	1.02 (1-1.05)	0.02	1.69 (0.72-3.96)	0.226
FAM3C	1.4 (0.71-2.74)	0.327	1.56 (0.83-2.92)	0.165	1.03 (1.01-1.05)	0.008	1.07 (0.44-2.58)	0.883
S100A12	1.07 (0.6-1.89)	0.829	0.51 (0.27-0.97)	0.04	1.03 (1.01-1.05)	0.005	2.61 (1.12-6.05)	0.026
FGF2	1.06 (0.55-2.02)	0.861	1.02 (0.54-1.93)	0.943	1.03 (1.01-1.06)	0.001	2.13 (0.88-5.14)	0.093

Table S3. Comparing mRNA expression levels for macrophage markers in normal brain tissue and tumor samples obtained from patients diagnosed with PDAC.

Genes identified in the Multivariate Cox proportional hazards model that showed prognostically relevant markers from examining TGFB2-Gene pairs that exhibited either significant increases in both TGFB2 and gene marker OS HR, or an increase in TGFB2 HR and a significant (P<0.05) interaction with the gene marker effect revealing 21 gene markers that impact OS in combination with TGFB2 levels. We also included known markers for classically characterized M1 (CD68) and M2 (MRC1/CD206) macrophages and TGFB2 for comparison.

	Normal Tissue (N=167)		Tumor Tissue (N=178)			
**Gene**	**Mean ± SEM**	**Median (Range)**	**Mean ± SEM**	**Median (Range)**	**Fold Change**	**P-value**
ADM	2.22 ± 0.13	2.31 (-9.97 - 6.39)	4.47 ± 0.12	4.43 (-1.15 - 7.98)	4.7	<0.0001
APOC1	2 ± 0.15	1.81 (-9.97 - 6.89)	7.52 ± 0.12	7.73 (2.96 - 12.19)	45.8	<0.0001
C1QA	3.21 ± 0.15	3.32 (-9.97 - 6.58)	7.29 ± 0.09	7.52 (3.45 - 10.54)	16.9	<0.0001
CCL18	-5.47 ± 0.26	-4.29 (-9.97 - 1.84)	4.41 ± 0.17	4.85 (-3.31 - 9.25)	944.8	<0.0001
CCL5	0.04 ± 0.12	0.1 (-9.97 - 3.99)	4.46 ± 0.1	4.53 (0.29 - 7.51)	21.4	<0.0001
CD40	3.07 ± 0.12	3.23 (-9.97 - 4.85)	4.3 ± 0.08	4.54 (-0.27 - 6.14)	2.4	<0.0001
CD68	1.87 ± 0.17	2.22 (-9.97 - 5.51)	7 ± 0.09	7.18 (2.41 - 9.4)	35.0	<0.0001
CD74	6.49 ± 0.16	6.59 (-9.97 - 9.69)	10.94 ± 0.08	11.15 (6.83 - 12.67)	21.8	<0.0001
EGLN3	0.64 ± 0.12	0.69 (-9.97 - 3.59)	4.8 ± 0.13	4.81 (-2.05 - 9.88)	17.8	<0.0001
HHLA2	-1.53 ± 0.12	-1.51 (-9.97 - 3.02)	2.32 ± 0.18	2.79 (-9.97 - 5.7)	14.5	<0.0001
HLA-DQB1	1.55 ± 0.15	1.7 (-3.63 - 10.02)	6.16 ± 0.14	6.41 (-0.43 - 9.38)	24.4	<0.0001
HLA-DRA	4.35 ± 0.15	4.41 (-9.97 - 7.64)	9.3 ± 0.1	9.51 (4.62 - 11.87)	31.0	<0.0001
HLA-DRB1	3.58 ± 0.09	3.35 (1.04 - 8.12)	7.98 ± 0.1	8.18 (2.93 - 10.9)	21.1	<0.0001
HLA-F	3.52 ± 0.13	3.52 (-9.97 - 7.33)	7.13 ± 0.07	7.21 (2.73 - 9.69)	12.3	<0.0001
IFI27	3.58 ± 0.13	3.66 (-9.97 - 8.36)	9.63 ± 0.12	9.78 (4.53 - 12.46)	66.3	<0.0001
IFNAR1	2.18 ± 0.11	2.35 (-9.97 - 3.68)	3.94 ± 0.04	3.94 (1.72 - 5.03)	3.4	<0.0001
IRF9	4.27 ± 0.14	4.38 (-9.97 - 7.12)	6.33 ± 0.06	6.39 (2.97 - 7.6)	4.2	<0.0001
LGALS9	3.26 ± 0.14	3.33 (-9.97 - 6.15)	7.3 ± 0.08	7.55 (2.59 - 9.1)	16.5	<0.0001
MARCO	-2.4 ± 0.21	-2.11 (-9.97 - 3.78)	3.16 ± 0.19	3.43 (-4.61 - 7.86)	47.0	<0.0001
MRC1	-0.24 ± 0.14	-0.03 (-9.97 - 3.43)	3.05 ± 0.13	3.1 (-2.05 - 6.32)	9.8	<0.0001
SPI1	1.13 ± 0.13	1.16 (-9.97 - 4.19)	4.46 ± 0.09	4.63 (0.85 - 7.14)	10.1	<0.0001
TGFB1	2.38 ± 0.13	2.4 (-9.97 - 5.83)	5.42 ± 0.06	5.65 (1.82 - 7.29)	8.3	<0.0001
TGFB2	-0.22 ± 0.12	-0.09 (-9.97 - 3.21)	2.76 ± 0.11	2.82 (-3.05 - 6.54)	7.9	<0.0001
TLR8	-4.21 ± 0.15	-4.04 (-9.97 - 0.67)	-0.61 ± 0.13	-0.47 (-6.51 - 3.89)	12.1	<0.0001

A

	PDAC patient sub-groups				G-test/Kruskal Wallis
	TGFB2^{high}/IRF9^{high}	TGFB2^{high}/IRF9^{low}	TGFB2^{low}/IRF9^{high}	TGFB2^{low}/IRF9^{low}	Pval
**Cancer Stage**					
Stage III/IV	1	1	1	5	0.179
Stage I/II	48	39	39	41	
**Histological Grade**					
Grade 1/2	30	26	29	40	0.042
Grade 3/4	19	14	10	7	
**Sex**					
Female	19	23	20	18	0.188
Male	30	17	20	30	0.188
**Age**					
Median (Range)	63 (35 - 84)	62.5 (40 - 88)	68.5 (48 - 85)	66 (39 - 81)	0.057
n	49	40	40	48	

B

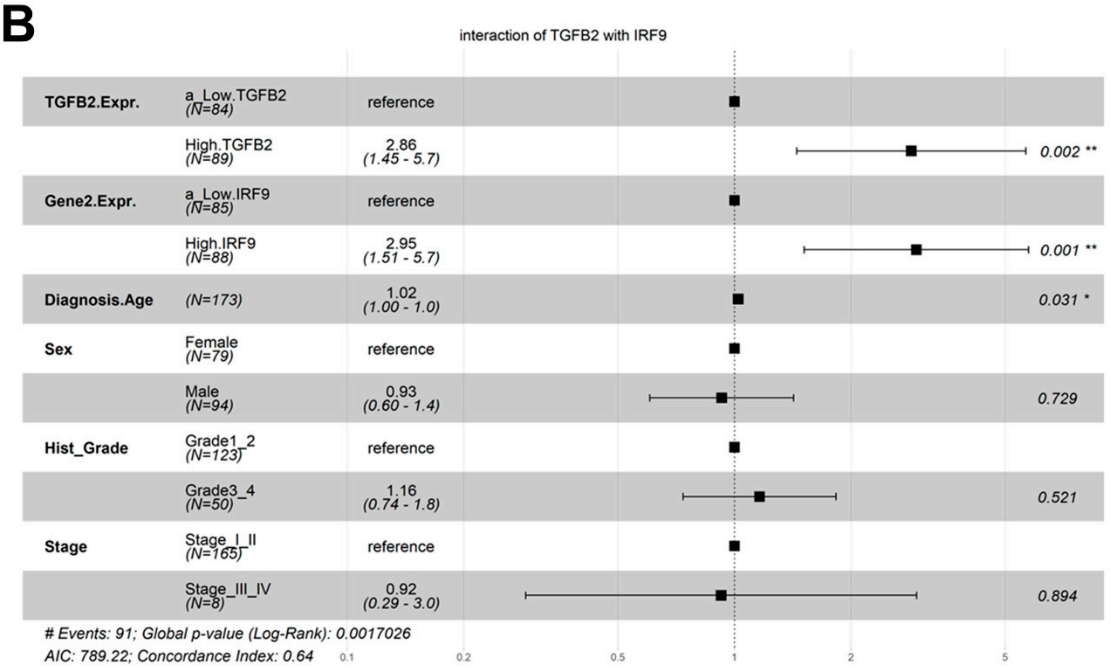

Figure S1. Multivariate Cox proportional hazards model to control for the confounding effect of age to investigate the impact of TGFB2 and IRF9 mRNA levels on OS.

[**A**] We compared the distribution of patient characteristics: Cancer Stage, Histological Grade, Sex, and Age, partitioned across four sub-groupings based on TGFB2 and IRF9 mRNA expression levels (high/low combinations; high cut-off at 50 percentile for the range of TPM values for the expression of each gene). A comparison of the four groups suggested a potential confounding impact of histological grade (Gtest of independence, P = 0.042) and age at diagnosis (P = 0.057) for PDAC patients. [**B**] Multivariate analyses were conducted to determine the impact of TGFB2 and IRF9 on the hazard ratio (HR) for OS outcome. The Cox proportional hazards model accounted for patient characteristics, and a TGFB2 x IRF9 interaction term factored into the model. OS outcomes exhibited the significant impacts of TGFB2mRNA levels (TGFB2^{high} HR (95% CI range) = 2.86 (1.45-5.65); P = 0.002), IRF9 mRNA levels (IRF9^{high} HR (95% CI range) = 2.95 (1.51-5.75); P = 0.001), Age at diagnosis (HR (95% CI range) = 1.02 (1-1.04); P = 0.031), and the interaction term (HR (95% CI range) = 0.32 (0.13-0.76); P = 0.01) in the model. Sex (P=0.7), Cancer Stage (P=0.9), and Histological Grade (P=0.5) were not significant factors impacting OS. Therefore, we only included age at diagnosis for patient characteristics in our investigations

A

	PDAC patient sub-groups				G-test/Kruskal Wallis
	TGFB2^{high}/IFI27^{high}	TGFB2^{high}/IFI27^{low}	TGFB2^{low}/IFI27^{high}	TGFB2^{low}/IFI27^{low}	P-val
**Cancer Stage**					
Stage III/IV	1	1	3	3	0.496
Stage I/II	48	39	37	43	
**Histological Grade**					
Grade 1/2	32	24	30	39	0.064
Grade 3/4	17	16	9	8	
**Sex**					
Female	18	24	18	20	0.157
Male	31	16	22	28	
**Age**					
Median (Range)	64 (35 - 84)	61 (40 - 88)	70.5 (48 - 82)	66 (39 - 85)	0.021
n	49	40	40	48	

B

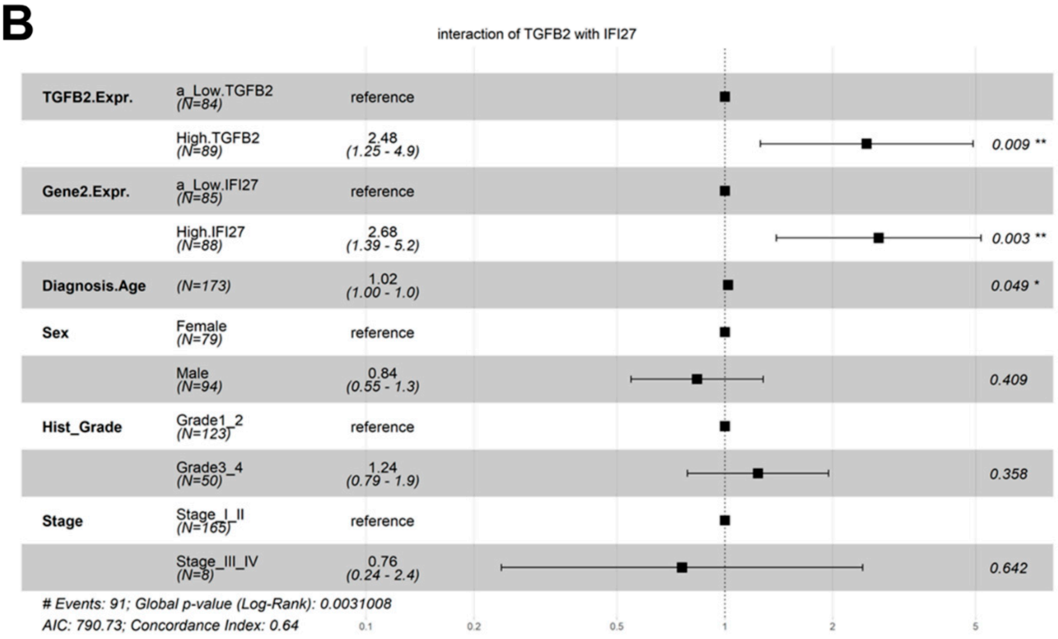

Figure S2. Multivariate Cox proportional hazards model to control for the confounding effect of age to investigate the impact of TGFB2 and IFI27 mRNA levels on OS.

[**A**] We compared the distribution of patient characteristics: Cancer Stage, Histological Grade, Sex, and Age, partitioned across four sub-groupings based on TGFB2 and IFI27 mRNA expression levels (high/low combinations; high cut-off at 50 percentile for the range of TPM values for the expression of each gene). A comparison across the four groups suggested a potential confounding impact of histological grade (Gtest of independence, P = 0.064) and age at diagnosis (P = 0.021) for PDAC patients. [**B**] Multivariate analyses were conducted to determine the impact of TGFB2 and IFI27 on the hazard ratio (HR) for OS outcome. The Cox proportional hazards model accounted for patient characteristics, and a TGFB2 x IFI27 interaction term factored into the model. OS outcomes exhibited the significant impacts of TGFB2mRNA levels (TGFB2^{high} HR (95% CI range) = 2.48 (1.25-4.92); P = 0.009), IFI27 mRNA levels (IFI27^{high} HR (95% CI range) = 2.68 (1.39-5.18); P = 0.003), Age at diagnosis (HR (95% CI range) = 1.02 (1-1.04); P = 0.049), and the interaction term (HR (95% CI range) = 0.41 (0.17-0.96); P = 0.041) in the model. Sex (P=0.4), Cancer Stage (P=0.6), and Histological Grade (P=0.4) were not significant factors impacting OS. Therefore, we only included age at diagnosis for patient characteristics in our investigations.

A

Variable	OS Hazard Ratio (95% CI)	Univariate P-value
Age	1.0279 (1.007-1.0492)	0.009
TGFB1	1.01 (0.67-1.54)	0.957
TGFB2	1.58 (1.03-2.43)	0.035
TGFB3	0.99 (0.66-1.5)	0.968
TGFBR1	1.45 (0.96-2.19)	0.08
TGFBR2	1.19 (0.79-1.8)	0.407
TGFBR3	0.9 (0.6-1.36)	0.631

B

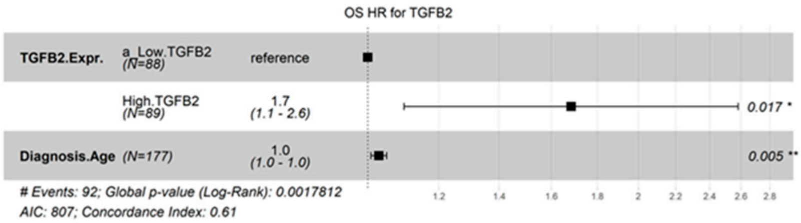

Figure S3. The prognostic impact of high levels of TGFB2 mRNA expression is independent of age in PDAC patients in both univariate and multivariate Cox proportional hazards models.

We implemented Univariate Cox proportional hazards models to assess the prognostic impact of TGFB ligands (TGFB1/2/3; N=177), receptors (TGFBR1/2; N=177), and Age (N=178) on the OS of PDAC patients. [**A**]. There were significant increases in HR for the TGFB2^{high} group of patients (HR (95% CI range) = 1.58 (1.03-2.43); P = 0.035) and age at diagnosis as a linear co-variate (HR (95% CI range) = 1.0279 (1.007-1.0492); P = 0.009). The prognostic impact for TGFBR1 showed an increase in HR that was borderline significant (HR (95% CI range) = 1.45 (0.96-2.19); P = 0.08). Patients with high levels of TGFB1, TGFB3, TGFBR2, and TGFBR3 mRNA did not significantly impact OS (HR ranged from 0.9 to 1.01). [**B**]. High levels of TGFB2 (greater than or equal to the median cut-off value) and Age as a linear co-variate indicated significant effects on OS outcomes in the univariate analysis and were further investigated in a multivariate model that included age as a confounding variable. Testing age at diagnosis as a linear covariate with TGFB2^{high} group of patients using the Multivariate Cox proportional hazards model showed the HR (95% CI) for TGFB2^{high} group of patients was 1.68 (1.10 - 2.58; P = 0.017). The effect of TGFB2 mRNA was independent of the confounding effect of age at diagnosis for this cohort of PDAC patients. Testing age at diagnosis as a linear covariate showed that this variable's HR (95% CI) was 1.029 (1.009 - 1.05; P = 0.0051).

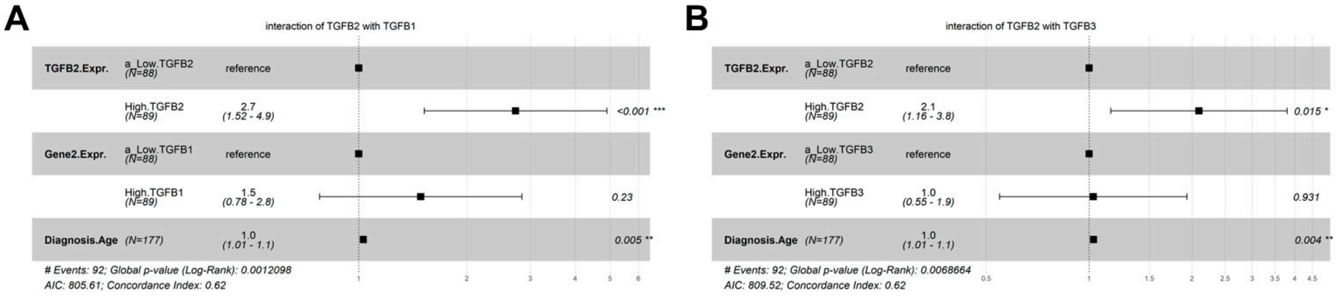

Figure S4. High TGFB2 mRNA expression in PDAC patients was independently associated with higher hazard ratios when age and interaction terms were considered in a Cox proportional hazards model investigating TGFB1 or TGFB3.

Multivariate analyses were conducted to determine the impact of TGFB2 and levels of other TGFB ligands, TGFB1 or TGFB2, on OS. The Cox proportional hazards model accounted for age at diagnosis and a TGFB2 x TGFB1/3 interaction term factored into the model. Three variables were considered to assess the independent effects of these variables: mRNA expression levels for TGFB2 and TGFB1/3 (high/low; high cut-off at 50 percentile for the range of TPM values) and age at diagnosis. Forest plots were used to visualize hazard ratios for OS outcomes. [**A**] Testing the effect of interaction between TGFB1 and TGFB2 showed that patients in theTGFB2^{high} group experienced a statistically significant increase in HR (HR (95% CI range) = 2.73 (1.52-4.9); P = 0.001). However, patients in theTGFB1^{high} group did not exhibit a significant increase in HR (HR (95% CI range) = 1.49 (0.78-2.84); P = 0.23) controlling for the diagnosis age group of patients (P = 0.005) and significant effect of the interaction term (HR (95% CI range) = 0.39 (0.18-0.85); P = 0.018). [**B**] A statistically significant increase in the hazard ratio (HR) was observed in patients with high TGFB2 expression (HR = 2.1, 95% CI 1.16-3.8, P = 0.015). However, in patients with high TGFB3 expression, no significant increase in HR was observed (HR = 1.03, 95% CI 0.55-1.93, P = 0.931) controlling for the significant effect of age at diagnosis (HR = 1.03, 95% CI 1.01-1.05, P = 0.004). Furthermore, no significant effect of the interaction term was observed (HR = 0.69, 95% CI 0.3-1.59, P = 0.381).

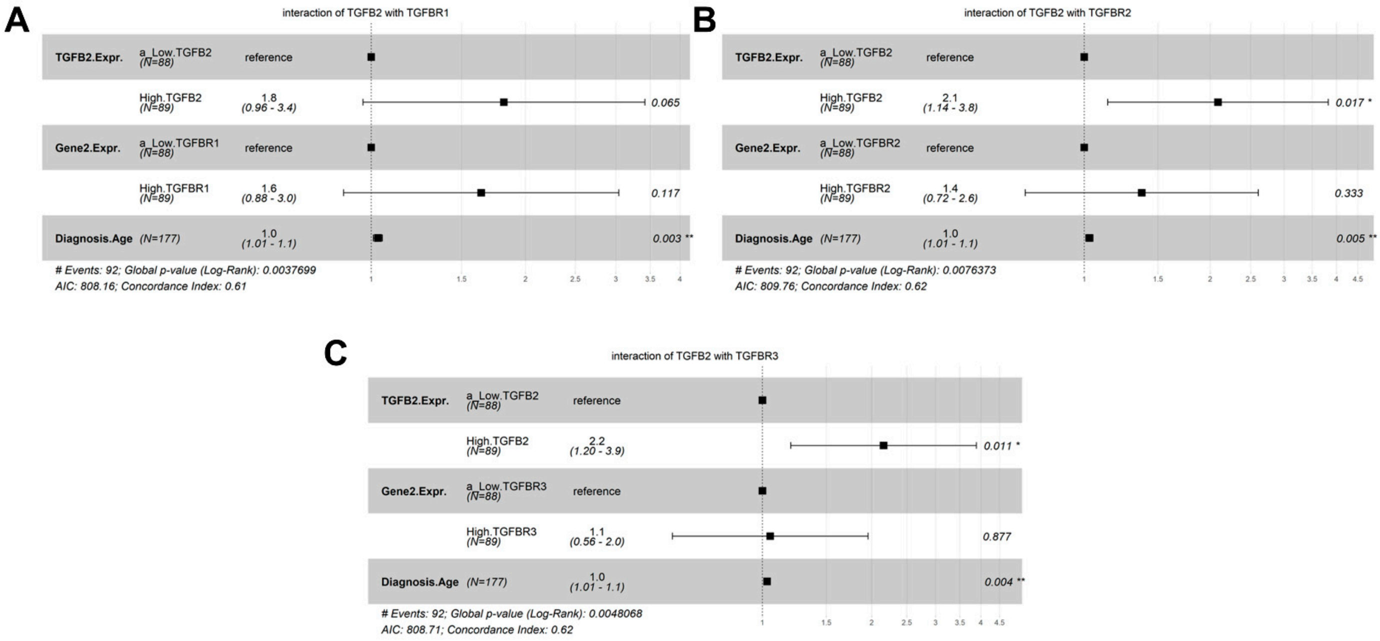

Figure S5. High TGFB2 mRNA expression in PDAC patients was independently associated with higher hazard ratios when age and interaction terms are considered in a Cox proportional hazards model investigating receptors for TGFB ligands.

Multivariate analyses were conducted to determine the impact of TGFB2 and TGFB receptors, TGFBR1/2/3 on OS. The Cox proportional hazards model accounted for age at diagnosis, and a TGFB2 x TGFBR1/2/3 interaction term factored into the model. Three variables were considered to assess the independent effects of these variables: mRNA expression levels for TGFB2 and TGFBR1/2/3 (high/low; high cut-off at 50th percentile for the range of TPM values) and age at diagnosis. Forest plots were used to visualize hazard ratios for OS outcomes. [**A**] The study's findings revealed an increase in the hazard ratio (HR) that was borderline not significant for patients with high expression of TGFB2. (HR (95% CI range) = 1.81 (0.96-3.42), P = 0.065). Similarly, the HR (95% CI range) was 1.64 (0.88-3.04) with a non-significant p-value of 0.117 for the patients with high expression of TGFBR1. However, a significant increase in HR was noted for factoring in age at diagnosis (HR (95% CI range) = 1.03 (1.01-1.05); P = 0.003) and no interaction effect (HR (95% CI range) = 0.74 (0.32-1.72), P = 0.485). [**B**] There was a significant increase in HR for TGFB2^{high} group of patients (HR (95% CI range) = 2.09 (1.14-3.83); P = 0.017), but not for TGFBR2^{high} group of patients (HR (95% CI range) = 1.37 (0.72-2.6); P = 0.333), accounting for a significant age effect (HR (95% CI range) = 1.03 (1.01-1.05); P = 0.005) and non-significant interaction term (HR (95% CI range) = 0.62 (0.26-1.45); P = 0.269). [**C**] Similarly, there was a significant increase in HR for TGFB2^{high} group of patients (HR (95% CI range) = 2.16 (1.2-3.89); P = 0.011) in the multivariate model that included TGFBR3 that showed a non-significant effect on HR forTGFBR3^{high} group of patients (HR (95% CI range) = 1.05 (0.56-1.95); P = 0.877), while taking into account a significant effect of age at diagnosis (HR (95% CI range) = 1.03 (1.01-1.05); P = 0.004) and a non-significant interaction effect (HR (95% CI range) = 0.62 (0.27-1.43); P = 0.263).

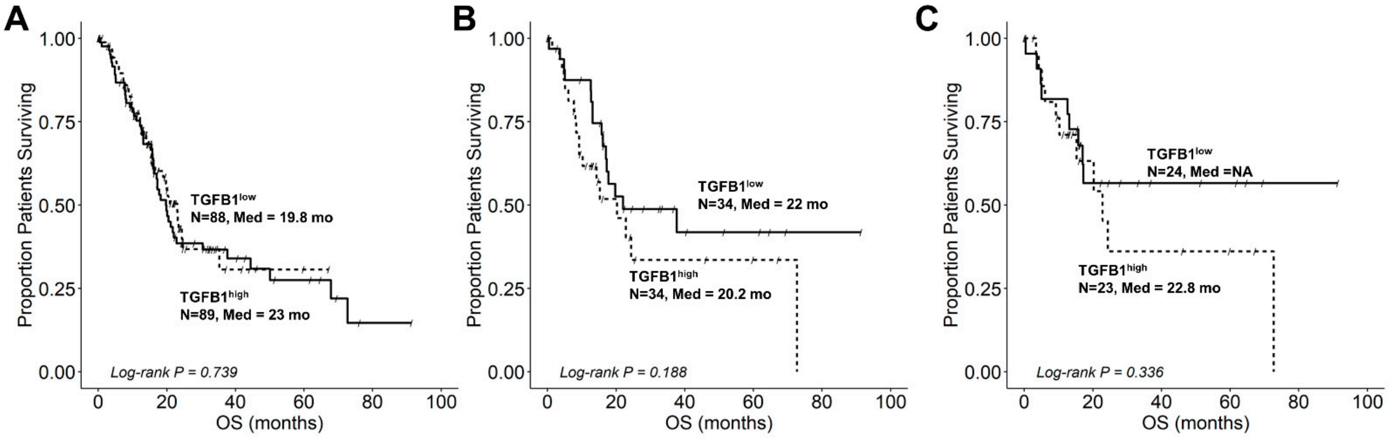

Figure S6. Prognostic impact of TGFB1 mRNA levels at low macrophage levels.

OS survival data correlated with mRNA expression for PDAC patients were depicted as Kaplan-Meier curves to determine the prognostic impact of TGFB1 median expression cut-off values for high versus low mRNA expression patient sub-groupings) for all PDAC patients (A. N=177), PDAC patients with low macrophage populations (B. N=68), and PDAC patients with low macrophage and low neoantigen levels (C. N=47) (http://kmplot.com/analysis/index.php?p=service&cancer=pancancer_rnaseq accessed 23rd May 2024). **A.** Comparing the median OS time for 88 patients from the TGFB2^{low} group (Median = 19.8 (95% CI: 16.2 - 37.7, Events = 50) months) with the TGFB2^{high} group (N=89, Median OS = 23 (95% CI: 18.9 - NA, Events = 42) months) showed that TGFB2 mRNA levels did not significantly impact OS (Log-rank Chi-Square = 0.111, P-value = 0.739). **B.** Examination of patients with low macrophage levels showed the median OS time for 34 TGFB2^{low} patients was 22 (95% CI: 17 - NA, Events = 16) months and was not significantly different from the TGFB2^{high} group of patients (N=34, Median = 20.2 (95% CI: 10.3 - NA, Events = 18) months; Log-rank Chi-Square = 1.73, P = 0.188). **C.** PDAC patients with low macrophage and neoantigen levels did not achieve statistical significance when comparing TGFB2^{low} (N=24, Median = NA, Events = 9) versus TGFB2^{high} (N=23, Median = 22.8 (95% CI: 15.3 - NA, Events = 11) months; Log-rank Chi-Square = 0.93, P-value = 0.336) groups.

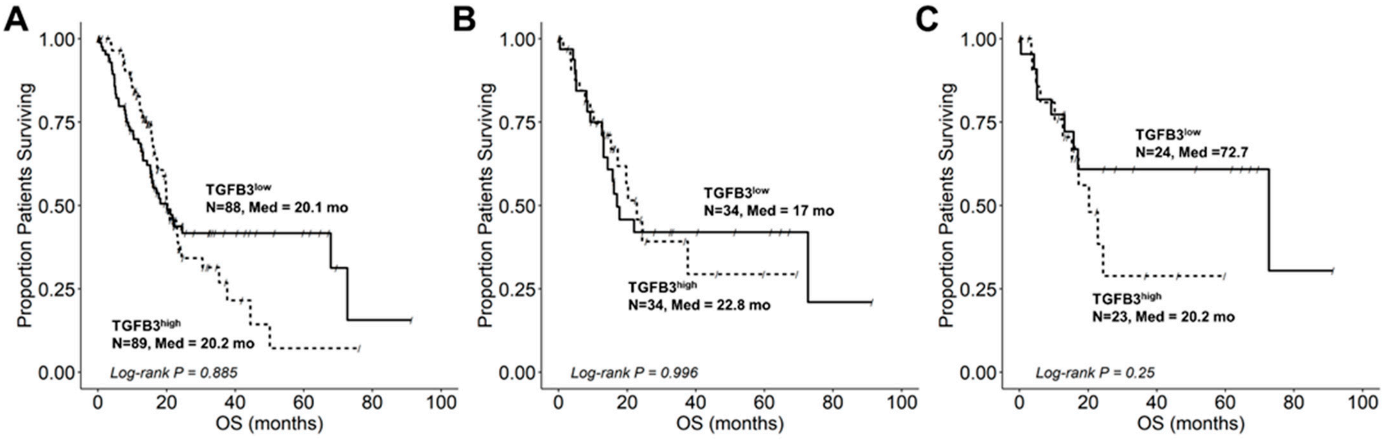

Figure S7. Prognostic impact of TGFB3 mRNA levels at low macrophage levels.

OS survival data correlated with mRNA expression for PDAC patients were depicted as Kaplan-Meier curves to determine the prognostic impact of TGFB3 median expression cut-off values for high versus low mRNA expression patient sub-groupings) for all PDAC patients (A. N=177), PDAC patients with low macrophage populations (B. N=68), and PDAC patients with low macrophage and low neoantigen levels (C. N=47) (http://kmplot.com/analysis/index.php?p=service&cancer=pancancer_rnaseq accessed 23rd May 2024). **A.** Comparing the median OS time for 88 patients from the TGFB2^{low} group (Median = 20.1 (95% CI: 15.33 - NA, Events = 46) months) with the TGFB2^{high} group (N=89, Median OS = 20.2333333 (95% CI: 18.9 - 24.4, Events = 46) months) showed that TGFB2 mRNA levels did not significantly impact OS (Log-rank Chi-Square = 0.0209612, P = 0.885). **B.** Examination of patients with low macrophage levels showed that the median OS time for 34 TGFB2^{low} patients was 17 (95% CI: 13.1 - NA, Events = 18) months and was not significantly different from that of the TGFB2^{high} group of patients (N=34, Median = 22.8 (95% CI: 17.3 - NA, Events = 16) months; P = 0.996). **C.** PDAC patients with low macrophage and neoantigen levels did not achieve statistical significance when comparing TGFB2^{low} (N=24, Median = 72.7 (95% CI: 15.8 - NA, Events = 9) months versus TGFB2^{high} (N=23,) Median = 20.2 (95% CI: 15.3 - NA, Events = 11) months; Log-rank Chi-Square = 1.32, P = 0.25) groups.

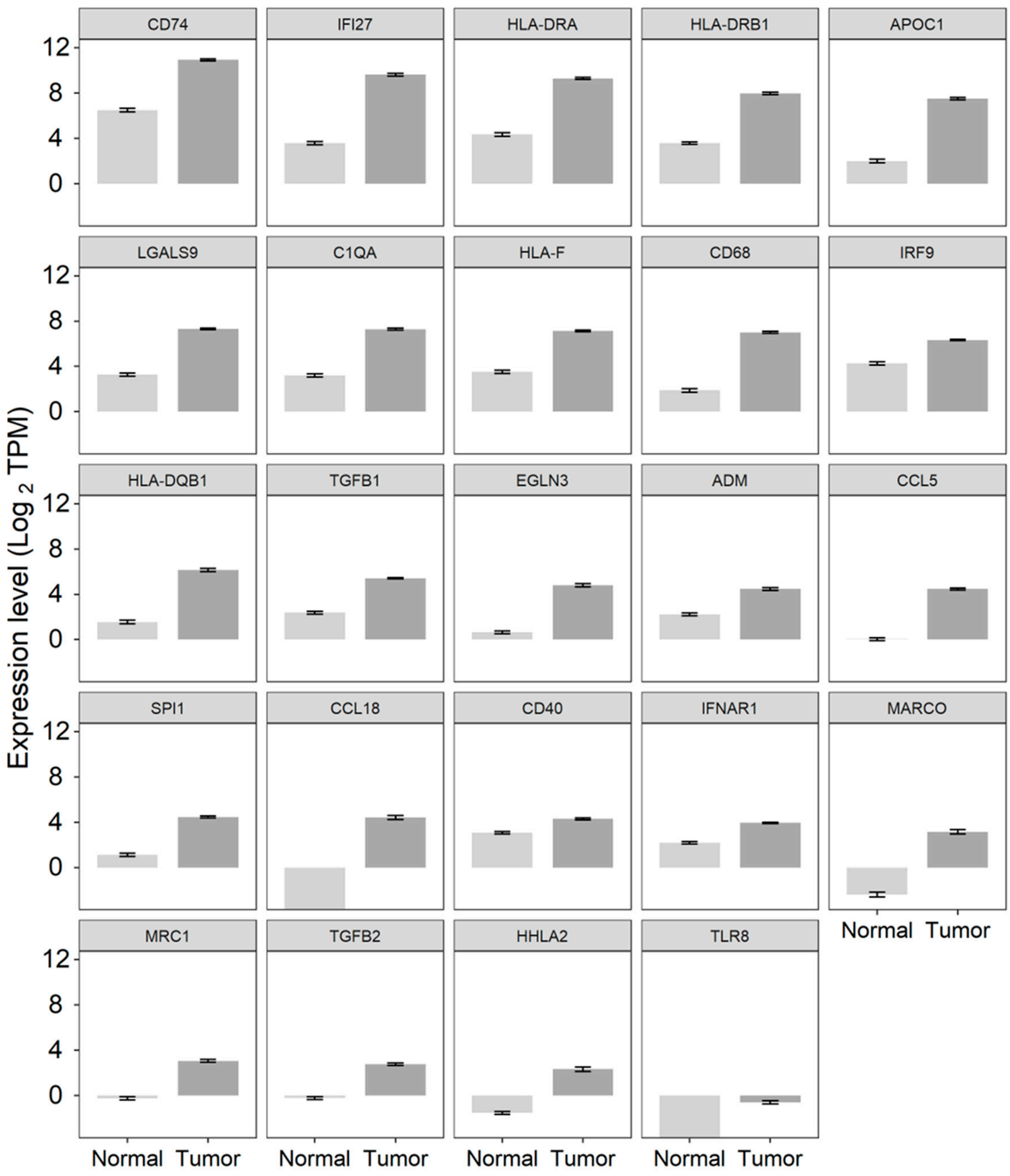

Figure S8. Comparison of normal versus tumor tissue samples for genes exhibiting significant prognostic impacts on OS from the multivariate Cox regression models

We evaluated the potential of select genes as biomarkers using a comparative analysis of mRNA levels for macrophage markers, including TGFB1, TGFB2, CD68, and MRC1, in both normal and tumor tissues obtained from the UCSC Xena web platform. The log2 transformed TPM values are presented as bar charts (mean ± SEM) between normal pancreatic tissue (N=167; depicted in light grey) and pancreatic cancer tissue samples (N=178; depicted in dark grey), ordered according to expression levels in tumor tissue samples. All 24 genes exhibited fold changes greater than 2 ($P<0.0001$ for all comparisons). Sixteen of the macrophage markers were upregulated greater than 10-fold in tumor compared to normal tissues: CCL18, MARCO, APOC1, CD68, HLA-DRA, HLA-DQB1, CD74, CCL5, HLA-DRB1, EGLN3, C1QA, LGALS9, HHLA2, HLA-F, TLR8, SPI1. Of these 16 genes, TLR8 was expressed at very low levels in tumor tissue (< 1 TPM). In normal tissues, TGFB2, MRC1, MARCO, CCL8, CCL5,and EGLN3 were expressed at very low levels. The highest levels of expression in tumor tissue were observed for CD74 (Mean ± SEM = 10.94 ± 0.08), IFI27 (Mean ± SEM = 9.63 ± 0.12), and HLA-DRA (9.3 ± 0.1).
